# Design parameter effects on controlled drug delivery through implantable hydrogels

**DOI:** 10.1007/s13346-026-02067-z

**Published:** 2026-02-05

**Authors:** Benita Jowell, Wenbo Zhan

**Affiliations:** School of Engineering, University of Aberdeen, Aberdeen, AB24 3UE UK

**Keywords:** Hydrogel, Cancer recurrence, Chemotherapy, Drug transport, Mathematical model

## Abstract

Glioblastoma recurrence after surgery is a major contributor to its high mortality, primarily occurring near the original tumour margin. Various hydrogels have been developed to fill the post-surgical cavity and deliver drugs to the surrounding brain tissue to eliminate residual cells. However, the impact of tissue, hydrogel, and drug properties on delivery outcomes remains unclear. Here, a parametric study is conducted to investigate these effects using mathematical modelling. The results show that post-surgical oedema strongly influences delivery: longer duration or delayed onset of oedema can homogenise drug distribution, with delayed onset yielding a larger and more sustained therapeutic drug volume. Hydrogels with higher permeability or lower drug affinity enhance early concentration and distribution but decline faster over time. Drugs with lower intracellular partitioning improve early efficacy, whereas those with stronger binding to cellular or extracellular components sustain delivery longer. Lower transvascular permeability and slower elimination further enhance outcomes, while extracellular diffusivity must be optimised to maximise drug concentration and distribution. These findings provide guidance for optimising hydrogel-based drug delivery systems to prevent glioblastoma recurrence.

**Graphical abstract:**

**Figure:**
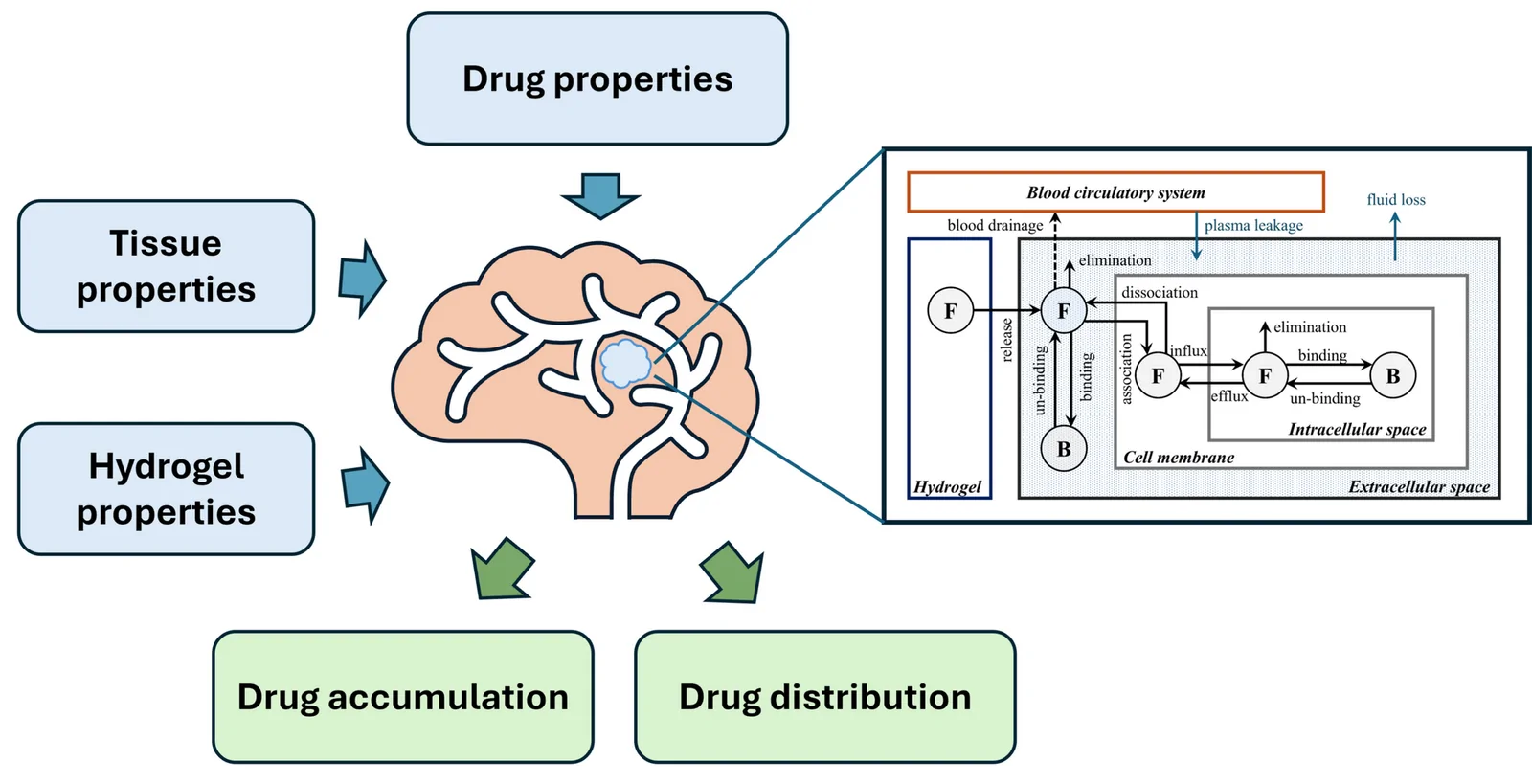

## Introduction

Glioblastoma is the most common and aggressive primary brain tumour in adults [1], posing a major challenge to global healthcare. Despite advances in modern medicine over recent decades, the median survival of patients remains limited to approximately 15 months [2]. Tumour recurrence within seven to nine months after surgery has been identified as one of the principal causes of treatment failure [3]. Clinical data indicate that over 90% of glioblastoma recurrences occur within 2 cm of the original tumour margin, where residual cancer cells survive initial surgery, radiotherapy, and chemotherapy [3].

Hydrogels have been engineered as local drug delivery systems with the aim of reducing glioblastoma recurrence. Following implantation into the surgical resection cavity, these hydrogels enable prolonged and spatially confined release of chemotherapeutic agents, often through nanocomposite or supramolecular designs that enhance drug stability, penetration, and retention within peri-tumoural brain tissue [4]. Beyond single-agent chemotherapy, increasing attention has been directed towards combination strategies, including multi-drug release platforms [5], chemo-immunomodulatory hydrogels [6], and stimuli-responsive systems [7–10] designed to overcome therapeutic resistance and remodel the post-surgical tumour microenvironment. Several recent studies have also focused on targeting glioma stem-like cells [11] and post-operative inflammation [12], recognising their central roles in glioblastoma recurrence. The therapeutic efficacy of these approaches critically depends on drug transport from the hydrogel into the adjacent brain tissue. This transport arises from multiple coupled physiological and physicochemical processes that are jointly governed by the properties of the brain tissue, the drug, and the hydrogel. Elucidating the influence of these parameters is therefore essential for the rational design and optimisation of hydrogel-based delivery systems. However, systematic experimental investigation of these effects requires a strictly controlled and reproducible drug delivery environment, which is difficult to achieve in vivo and is often economically impractical due to the complexity and dynamic nature of the physiological milieu.

Mathematical modelling offers an alternative and complementary approach [13–15]. By employing governing equations to describe the coupled transport processes, such models enable parametric studies in which individual properties can be systematically varied within a reproducible intratumoural environment, allowing their specific impacts to be isolated and quantified [16]. This modelling framework was originally developed to investigate antibody delivery in idealised solid tumours [17–19] and was subsequently adapted for drug delivery applications across a range of tumour types [20–25]. In particular, it was tailored to simulate the release of chemotherapeutic agents from polymeric gels into brain tissue and validated against experimentally measured drug concentration profiles [26–28]. The framework was later extended to analyse the delivery of different chemotherapeutic drugs via polymer-based wafers and to examine the influence of interstitial fluid flow on delivery performance [29, 30]. Our recent studies have further applied this framework to evaluate the transport behaviour of several chemotherapeutic agents [31]. However, the influence of key system properties has not yet been comprehensively investigated.

The present study is aimed to comprehensively evaluate the impact of multiple key properties on hydrogel-mediated drug delivery for the prevention of glioblastoma recurrence after surgery through computational simulation. The mathematical model incorporates essential physiological and physicochemical processes, including interstitial fluid flow associated with the progression of oedema; drug diffusion within the hydrogel and into the surrounding brain tissue; convective and diffusive transport within the brain tissue; drug clearance via vascular drainage; degradation and bioreactive processes; and cellular internalisation. Delivery performance is quantified in terms of total drug accumulation, spatial distribution uniformity, and therapeutic drug distribution volume. A generic hydrogel-drug system is considered without specifying a particular formulation or drug type, thereby enabling exploratory parametric studies to systematically examine the influence of a wide range of hydrogel and drug properties.

## Methods and materials

### Mathematical model

The mathematical model at the macroscale is developed by assuming that the blood capillaries are distributed throughout the brain tissue, which is treated as a porous medium [17]. This assumption is based on the fact that the intercapillary distance is typically two to three orders of magnitude smaller than the characteristic dimension of the brain. The model comprises the continuity and momentum conservation equations governing interstitial fluid flow, together with mass transfer equations describing drug transport within the implanted hydrogel and into the surrounding brain tissue. The detailed formulation of these equations, along with validation against experimental data, has been reported in our previous study [31]. The governing equation and the results of the validation study are presented in Appendix 1, Table 2, Fig. 16.

**Fig. 1:**
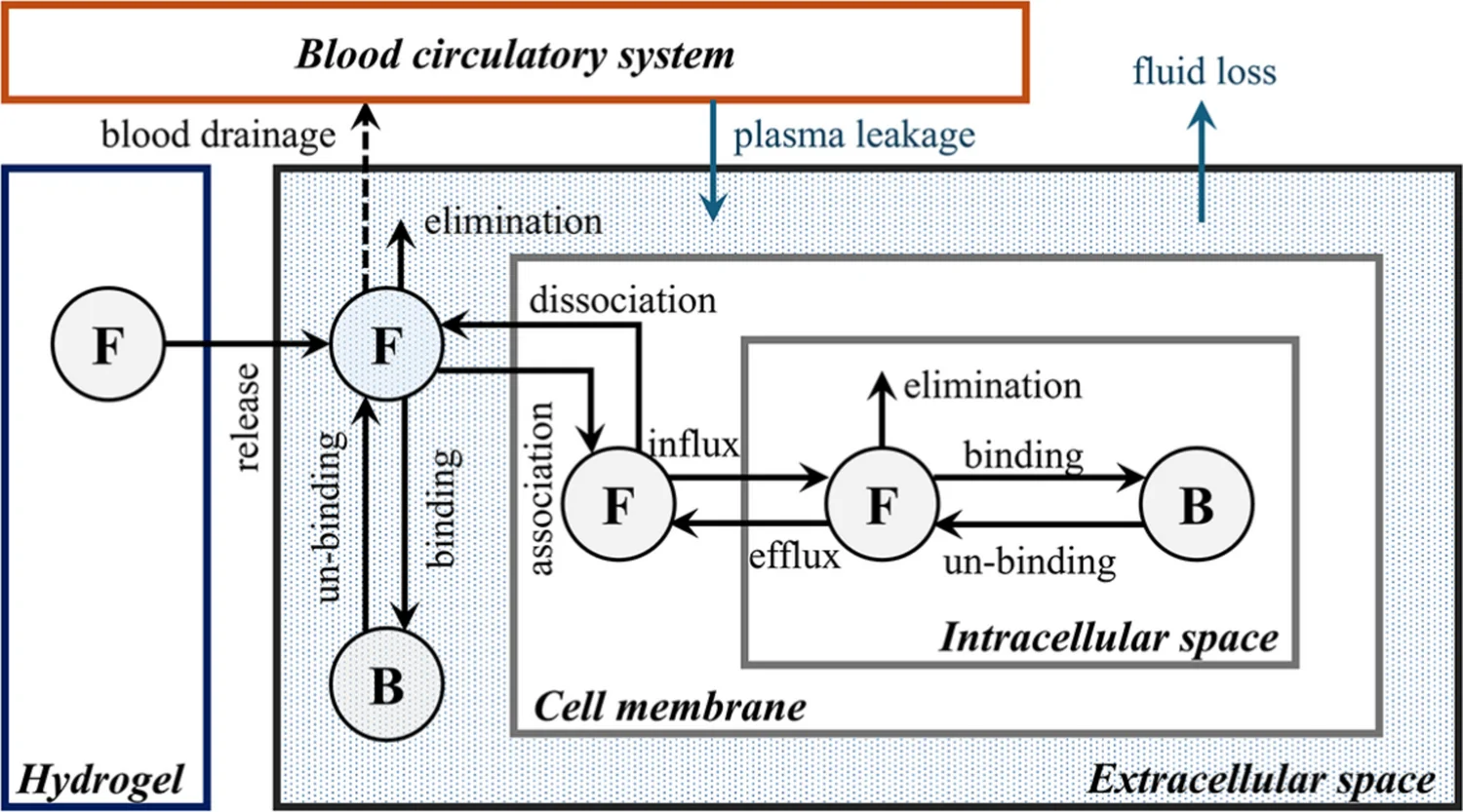
Schematic representation of fluid and drug transport within the brain tissue. The letters F and B indicate the drug in its free and bound forms, respectively. Black arrows represent drug transport, while blue arrows denote fluid flow. Drug transport from the tissue to the blood is governed by the drug’s ability to penetrate the blood–brain barrier, which is shown as the dashed line

Specifically, plasma extravasates from the blood vessels into the brain parenchyma as interstitial fluid, driven by the transvascular pressure gradient, and is eventually cleared at the brain surface and ventricles. This leakage may be further exacerbated by surgery-induced tissue damage, which in turn leads to oedema. The loaded drug diffuses from the hydrogel into the brain tissue, where its transport is governed by diffusion arising from the concentration gradient and convection associated with interstitial fluid flow. Moreover, the drug can be internalised by cells, bind to proteins, or be eliminated through physical degradation, enzyme-mediated reactions, or clearance to the bloodstream. These processes are schematically presented in Fig. 1.

### Model geometry

**Fig. 2:**
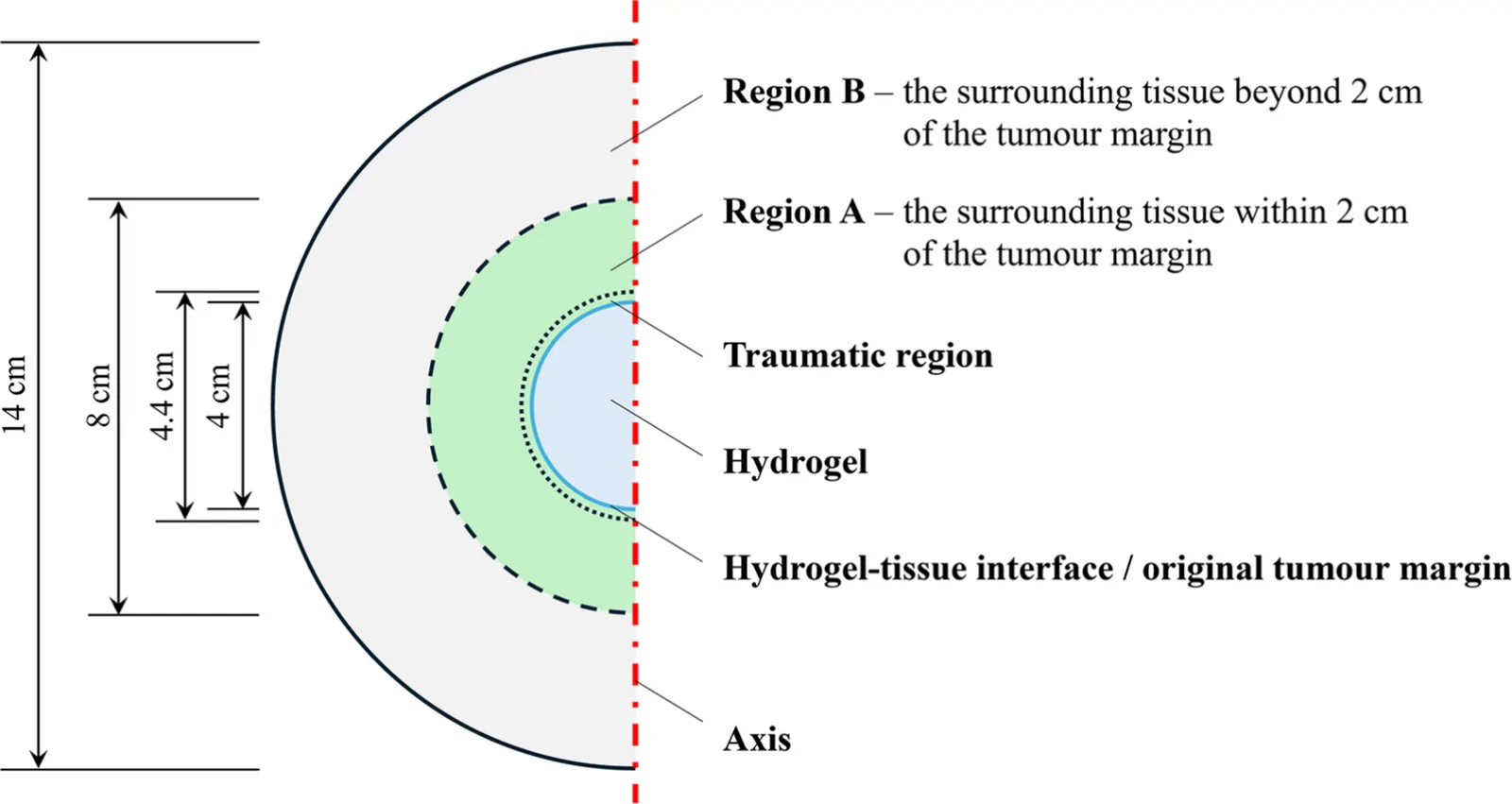
Model geometry. The 2-D axisymmetric configuration comprises the hydrogel region (light blue) and the surrounding tissues, which are divided into Region A (light green) and Region B (light grey), corresponding to tissues within and beyond 2 cm of the original tumour margin, respectively. Their interface is indicated by the dashed line, while the boundary traumatic region is highlighted with a dotted line

The numerical simulation of hydrogel-mediated delivery is performed in a 2-D axisymmetric configuration, as depicted in Fig. 2. The radius of the tissue is 7 cm, which matches the general brain dimension. The surgery-left cavity is filled by the drug-loaded hydrogels; its radius is 2 cm, which is consistent with the dimension of the cavity from the patient-specific model [31]. The traumatic layer surrounds the hydrogels and has a thickness of 2 mm [32]. The final computational mesh generated in COMSOL Multiphysics consists of approximately 30,000 triangular elements after conducting a mesh independence test. The finest elements in 0.3 mm are imposed on the interface between the hydrogels and traumatic tissue for high resolution of numerical solutions.

### Model parameters

The parameters representing tissue properties, along with the corresponding sources, are summarised in Table 1. The rationale for selecting the baseline values and the range of each key property is justified as follows.

**Table 1:** Model parameters of tissue property

Symbol	Parameter	Unit	Value	Source
α	Volume fraction of tissue extracellular space	-	0.2	[27, 29]
γ	Volume fraction of intracellular space	-	0.65	[27, 29]
ρ	Density of the interstitial fluid	$\mathrm{kg}/{\mathrm{m}}^{3}$	$1.0\times {10}^{3}$	[24, 29, 33]
μ	Dynamic viscosity of the interstitial fluid	$\mathrm{Pa}\bullet \mathrm{s}$	$7.8\times {10}^{-4}$	[24, 33]
$\Delta \pi$	Osmotic pressure difference between the blood and interstitial fluid	$\mathrm{Pa}$	$2.7\times {10}^{3}$	[29, 34]
${p}_{\mathrm{b}}$	Blood pressure	$\mathrm{Pa}$	$4.6\times {10}^{3}$	[29, 34]
S/V	Area of blood vessel wall per tissue volume	${\mathrm{m}}^{-1}$	$7.0\times {10}^{3}$	[29, 34]
σ	Reflection coefficient for blood proteins	-	$9.1\times {10}^{-1}$	[34]
${L}_{0}$	Initial hydraulic conductivity of blood vessel walls	$\mathrm{m}/\mathrm{Pa}/\mathrm{s}$	$1.4\times {10}^{-13}$	[29]
κ	Tissue hydraulic conductivity	${\mathrm{m}}^{2}$	$7.1\times {10}^{-15}$	[29]

#### Oedema progression

Surgical resection of brain tumours can significantly damage the surrounding tissue, disrupt the local blood–brain barrier and allow increased plasma leakage from the vasculature into this traumatic tissue region, thereby leading to oedema formation. The following model [31, 35], developed based on experimental data [36, 37], describes the temporal changes in the hydraulic conductivity of blood vessels (L) within the traumatic tissue region, as a function of time

1 $$L=\left\{\begin{array}{ll}{L}_{0}& \left(t\le 6\; \mathrm{hours}\right)\\ {L}_{0}\left(4.583\times {10}^{-3}t-98\right)& \left(6\; \mathrm{hours}<t\le 12\; \mathrm{hours}\right)\\ 100{L}_{0}& \left(12\; \mathrm{hours}<t\le 24\; \mathrm{hours}\right)\\ {L}_{0}\left(-1.91\times {10}^{-4}t+116.5\right)& \left(24\; \mathrm{hours}<t\le 7\; \mathrm{days}\right)\\ {L}_{0}& \left(7\text{ days}<t\right)\end{array}\right.$$

where ${L}_{0}$ is the initial hydraulic conductivity of the blood vessel wall, and t is time.

Specifically, oedema was observed to occur approximately six hours after surgery in clinical practice, causing the hydraulic conductivity (L) to increase up to 100-fold compared with its baseline value [36, 37]. Therefore, the onset time (${O}_{\mathrm{ST}}$) and the degree of increase (${O}_{\mathrm{DG}}$) are set to 6 h and 100, respectively, while the duration of oedema (${O}_{\mathrm{PR}}$) is ideally set to 12 h. However, it is important to note that the progression of oedema can vary significantly among patients. To examine the effects of oedema progression, the parameters ${O}_{\mathrm{ST}}$ and ${O}_{\mathrm{DG}}$ are varied from 0 to 24 h and from 1 to 1000, respectively, while ${O}_{\mathrm{PR}}$ ranges from 3 to 48 h in the parametric study.

#### Drug diffusion coefficients

Diffusivity characterises the capacity of drug molecules to undergo thermal motion driven by concentration gradients. It is governed by both molecular attributes, including molecular weight, hydrophobicity/hydrophilicity, and surface charge, and the physicochemical properties of the transport medium, such as composition and porosity. The diffusivity of doxorubicin has been reported to range from $2.6\times {10}^{-10} {\mathrm{m}}^{2}/\mathrm{s}$ to $5.0\times {10}^{-10} {\mathrm{m}}^{2}/\mathrm{s}$ in various agarose gels [38]. Corresponding values for 5-fluorouracil and its hydrophobic analog in PEG hydrogels were $6.8\times {10}^{-10} {\mathrm{m}}^{2}/\mathrm{s}$ and $3.3\times {10}^{-10} {\mathrm{m}}^{2}/\mathrm{s}$, respectively [39]. Methotrexate exhibited diffusivities of $3.6\times {10}^{-10} {\mathrm{m}}^{2}/\mathrm{s}$ in 2% Carbopol gel and $1.0\times {10}^{-10} {\mathrm{m}}^{2}/\mathrm{s}$ in 25% PF-127 gel [40]. In brain tissue, the diffusivities of fluorouracil and temozolomide were reported as $1.2\times {10}^{-9} {\mathrm{m}}^{2}/\mathrm{s}$ [29] and $3.4\times {10}^{-10} {\mathrm{m}}^{2}/\mathrm{s}$ [41], respectively, whereas paclitaxel exhibited a reduced diffusivity of $9.0\times {10}^{-10} {\mathrm{m}}^{2}/\mathrm{s}$ [28, 29]. Taken together, these studies suggest a literature-based diffusivity range of approximately from $1.0\times {10}^{-10} {\mathrm{m}}^{2}/\mathrm{s}$ to $1.2\times {10}^{-9} {\mathrm{m}}^{2}/\mathrm{s}$. Given that hydrogels are commonly engineered to replicate the mechanical characteristics of biological tissues, a broad diffusivity range of $1.0\times {10}^{-11}-1.0\times {10}^{-7}{\mathrm{m}}^{2}/\mathrm{s}$ is adopted for both hydrogels (${D}_{\mathrm{F},\mathrm{HG}}$) and brain tissue (${D}_{\mathrm{F},\mathrm{ECS}}$) to systematically examine their impacts, respectively. The baseline value is set as $1.0\times {10}^{-9}{\mathrm{m}}^{2}/\mathrm{s}$ for both diffusion coefficients.

#### Drug partition coefficients

The partition coefficient is defined as the ratio of drug concentrations in two adjacent media at dynamic equilibrium. Its value depends on both the physicochemical properties of the drug and the characteristics of the two media.

In biological systems, the tissue extracellular space is typically considered an aqueous phase, whereas the cell membrane represents a lipid-rich, hydrophobic phase. Owing to the distinct hydrophilicity and hydrophobicity of each drug, their partitioning between the extracellular space and the cell membrane varies during transport from the extracellular milieu into the cell interior. The parameter ${P}_{\mathrm{ME}}$ is the ratio of drug mass in the extracellular space to that on the cell membrane at equilibrium and is commonly estimated experimentally via the drug’s partition coefficient between water and octanol. Reported values include 0.006 for cisplatin [42], 0.1 for 5-fluorouracil [26], 10.3 for carmustine [27], and 3162.3 for paclitaxel [28]. Accordingly, a range of 0—10,000 is adopted in the present study, with the baseline value of 1.0.

The partition coefficient between the intracellular and extracellular spaces (${P}_{\mathrm{IE}}$) is often assumed to be 1.0, as both compartments are generally aqueous [27]. However, differences in composition and solute concentrations between these spaces can lead to deviations from unity. In the absence of experimental measurements, this parameter is varied artificially from 0 to 100, and the baseline is 1.0.

The partition coefficient of drugs between the hydrogel and the surrounding brain tissue (${P}_{\mathrm{HG}}$) is typically assumed to be 1.0 [31], as hydrogels are usually treated as aqueous phases comparable to brain tissue. To account for potential variability, the partition coefficient is considered in the range 0–100, with 0 representing an extreme case in which no drug partitions into the brain tissue. The baseline is set to be 1.0.

#### Drug removal rates

The blood–brain barrier restricts transvascular transport for more than 98% of small-molecule drugs. Nevertheless, certain compounds, such as carmustine and paclitaxel, are capable of permeating the cerebral capillary wall. The rate parameter (${k}_{\mathrm{b}}$) represents the rate at which drugs transfer into the bloodstream. Reported values are $1.4\times {10}^{-2} {\mathrm{s}}^{-1}$ for carmustine [29] and $1.4\times {10}^{-4} {\mathrm{s}}^{-1}$ for paclitaxel [29], while temozolomide exhibits a rate of $1.1\times {10}^{-4} {\mathrm{s}}^{-1}$ [41]. The rate of cisplatin was reported to be $1.9\times {10}^{-4} {\mathrm{s}}^{-1}$ [26]. Accordingly, a broad range of $0-1.0 {\mathrm{s}}^{-1}$ is employed in the present study. The baseline value of this rate is $1.0\times {10}^{-6} {\mathrm{s}}^{-1}$.

Drugs distributed within brain tissue are also subject to physical degradation and biochemical reactions, both of which substantially affect local drug accumulation and penetration depth from the implanted hydrogel into the surrounding tissue. The elimination rate (${k}_{\mathrm{e}}$) varies widely among drugs, primarily due to differences in molecular structure. Reported elimination rates include $8.3\times {10}^{-5}{\text{ s}}^{-1}$ for carmustine [26], $6.8\times {10}^{-7} {\mathrm{s}}^{-1}$ for paclitaxel [29], and $5.6\times {10}^{-4}$ ${\mathrm{s}}^{-1}$ for 5-fluorouracil [29]. Doxorubicin presents a rate of $5.8\times {10}^{-4}$ ${\mathrm{s}}^{-1}$ [24, 43]. Consequently, a range of $1.0\times {10}^{-7}-1.0\times {10}^{-3} {\mathrm{s}}^{-1}$ is adopted in this study to capture the variability among different compounds, while the baseline is $1.0\times {10}^{-5} {\mathrm{s}}^{-1}$.

#### Drug binding coefficients

Drugs can reversibly bind to components of the extracellular matrix during transport through the interstitial spaces between cells. The ratio of the concentration of bound drug to that of the free drug is defined as the binding ratio in the extracellular space (${K}_{\mathrm{ECS}}$). Reported values include 4.4 for cisplatin [44], 5.0 for carmustine [27, 29], 0.18 for temozolomide [41], and 0.7 for methotrexate [45], whereas large biomolecules such as immunoglobulin G are generally assumed to exhibit negligible binding [26]. Accordingly, a range of 0–10, with the baseline of 1.0, is adopted in this study to evaluate the influence of this parameter.

Drugs may also bind to intracellular components following cellular uptake. The ratio of bound to free drug concentrations within the cell interior is defined as the intracellular binding ratio (${K}_{\mathrm{ICS}}$). In the absence of direct experimental measurements, this parameter is typically assumed to be equivalent to that in the extracellular space in previous modelling studies of drug delivery [29, 30]. To assess the sensitivity of model outcomes to this factor, the intracellular binding ratio is also varied within the range of 0–10 in the present parametric analysis. The baseline of this parameter is 1.0.

### Boundary conditions

A pressure of 580 Pa is applied at the outer surface of the computational domain, chosen to approximately represent the brain surface pressure of 657.9 Pa [46] and the ventricular surface pressure of 500 Pa [47]. The drug concentration at these surfaces is set to zero, as they border the meningeal and extracranial lymphatics and the circulating cerebrospinal fluid, which act as sinks for drug transport. The hydrogel surface is treated as a rigid wall on which the interstitial fluid flow has zero velocity, while the drug concentration at the hydrogel-tissue interface follows

2 $$\begin{array}{cc}{C}_{\mathrm{F},\mathrm{HG}}={P}_{\mathrm{HG}}{C}_{\mathrm{F},\mathrm{ECS}};& -{D}_{\mathrm{F},\mathrm{HG}}\frac{\partial {C}_{\mathrm{F},\mathrm{HG}}}{\partial n}=-{D}_{\mathrm{F},\mathrm{ECS}}\frac{\partial {C}_{\mathrm{F},\mathrm{ECS}}}{\partial n}\end{array}$$

where n is the normal direction of the interface. ${P}_{\mathrm{HG}}$ is the partitioning coefficient of the loaded drug between the hydrogel and its surrounding brain tissues.

### Quantification of delivery outcomes

The outcomes of hydrogel-mediated delivery under different delivery conditions are evaluated by the following indicators for cross-comparisons.

#### Normalised spatially averaged drug concentration

Drug availability in the brain tissue can be represented by the normalised spatially averaged concentration of free drugs in the tissue extracellular space (${\overline{C} }_{\mathrm{F},\mathrm{ECS}}$). It is defined as the spatially averaged concentration normalised by the initial free drug concentration in the hydrogel (${C}_{\mathrm{F},\mathrm{HG},0}$). This indicator is defined as

3 $${\overline{C} }_{\mathrm{F},\mathrm{ECS}}=\frac{\sum {C}_{\mathrm{F},\mathrm{ECS},\mathrm{i}}{V}_{\mathrm{i}}}{\sum {V}_{\mathrm{i}}{C}_{\mathrm{F},\mathrm{HG},0}}=\frac{\sum {C}_{\mathrm{F},\mathrm{ECS},\mathrm{i}}{V}_{\mathrm{i}}}{{V}_{\mathrm{Tissue}}{C}_{\mathrm{F},\mathrm{HG},0}}$$

where ${C}_{\mathrm{F},\mathrm{ECS},\mathrm{i}}$ is the local drug concentration. ${V}_{\mathrm{i}}$ is the local tissue volume, respectively, which ${V}_{\mathrm{Tissue}}$ refers to the total volume of the tissue.

#### Distribution non-uniformity

The extent of heterogeneous drug distribution can be quantified using the dimensionless distribution non-uniformity number ($\mathrm{NUN}$), as

4 $$\mathrm{NUN}=\frac{\sum \left|{C}_{\mathrm{F},\mathrm{ECS},\mathrm{i}}-{\overline{C} }_{\mathrm{F},\mathrm{ECS}}\right|{V}_{\mathrm{i}}}{{\overline{C} }_{\mathrm{F},\mathrm{ECS}}\sum {V}_{\mathrm{i}}}=\frac{\sum \left|{C}_{\mathrm{F},\mathrm{ECS},\mathrm{i}}-{\overline{C} }_{\mathrm{F},\mathrm{ECS}}\right|{V}_{\mathrm{i}}}{{\overline{C} }_{\mathrm{F},\mathrm{ECS}}{V}_{\mathrm{Tissue}}}$$

A lower NUN indicates a more homogeneous spatial distribution.

#### Drug coverage volume

The tissue volume in which the local drug concentration exceeds a specified threshold can be used to evaluate drug coverage for therapy under different delivery scenarios. In this study, the drug coverage volume is calculated as the total tissue volume where the local drug concentration exceeds 0.01% of the initial free drug concentration in the hydrogel, as

5 $$\begin{array}{cc}{V}_{\mathrm{cover}}=\sum {V}_{\mathrm{i}}& \left({C}_{\mathrm{F},\mathrm{ECS},\mathrm{i}}\ge 0.01\%{C}_{\mathrm{F},\mathrm{HG},0}\right)\end{array}$$

This threshold is selected based on a representative anti-brain cancer drug, carmustine, for which an extracellular concentration corresponding to 0.01% of its concentration [28] in the commercialised wafer [48] is sufficient to kill more than 90% of local tumour cells.

### Parameter sensitivity analysis

The response of delivery outcomes to each tissue and drug property is quantified using a sensitivity index S, defined as

6 $${S}_{\mathrm{i}}\left({t}_{\mathrm{j}}\right)=\frac{y\left({X}_{\mathrm{i}},{t}_{\mathrm{j}}\right)-y\left({X}_{\mathrm{ref}},{t}_{\mathrm{j}}\right)}{{X}_{\mathrm{i}}-{X}_{\mathrm{ref}}}\frac{{X}_{\mathrm{ref}}}{y\left({X}_{\mathrm{ref}},{t}_{\mathrm{j}}\right)}$$

where y denotes the delivery outcomes, including drug concentration, distribution non-uniformity, and therapeutic distribution volume. ${X}_{\mathrm{i}}$ represents the examined factor, while ${X}_{\mathrm{ref}}$ denotes the reference value corresponding to the baseline case in this study. The overall sensitivity index of a given factor over the studied period T is then calculated as

7 $${S}_{\mathrm{overall},\mathrm{i}}={\int }_{0}^{T}{S}_{\mathrm{i}}\left({t}_{\mathrm{j}}\right)dt/T$$

To enable direct comparison of the relative importance of different factors, the overall sensitivity indices are further normalised.

8 $${\overline{S} }_{\mathrm{overall},\mathrm{i}}=\frac{{S}_{\mathrm{overall},\mathrm{i}}}{\mathrm{max}\left({S}_{\mathrm{overall},1 }, {S}_{\mathrm{overall},2}, \cdots , {S}_{\mathrm{overall},\mathrm{N}}\right)}$$

where N is the number of examined influential factors.

## Results

In the parametric analysis, each parameter is independently varied within its prescribed range, while all other parameters are held constant at their baseline values.

### Impact of oedema progression

**Fig. 3:**
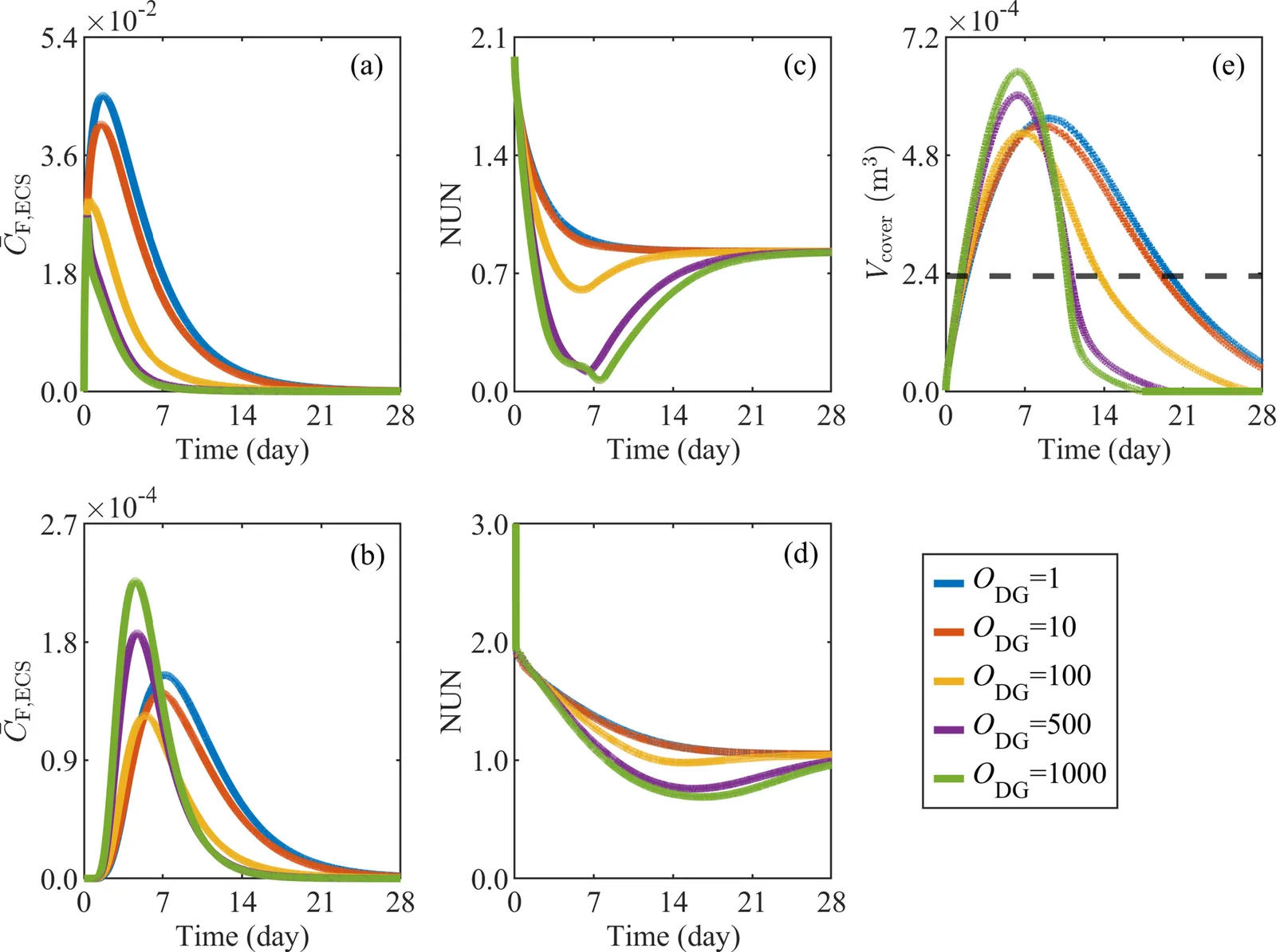
Comparison of drug delivery outcomes under different hydraulic conductivities of traumatic blood capillaries. Subplots show the time course of the averaged drug concentration in Region A (**a**) and Region B (**b**); the NUN in Region A (**c**) and Region B (**d**); and the total drug-covered volume (**e**). The dark grey dashed line in subplot (e) indicates the volume of the tissue region within 2 cm of the tumour. The initial NUN in Region B can be high and fluctuating due to the extremely low concentration and heterogeneous distribution. The y-axis scale is adjusted to highlight the period when the NUN becomes relatively stable

Figure 3 illustrates the effect of increased hydraulic conductivity of blood capillary walls on drug delivery outcomes. As shown in Fig. 3(a), the drug concentration in Region A exhibits a lower peak and a faster decline as the vessel wall conductivity in the traumatic region increases. This results from enhanced capillary leakage, which facilitates plasma extravasation into the surrounding tissue and strengthens interstitial fluid flow from the tumour margin towards the brain boundary, thereby promoting convective drug transport in the same direction. Although the accelerated flow increases the drug release rate from the hydrogel, it also shortens the duration of release, contributing to a lower peak and a more rapid decline in average concentration. Moreover, the intensified flow allows deeper penetration and yields a more uniform distribution, as shown in Fig. 3(c), compared with the gradual decay observed when oedema is absent.

In contrast, Region B exhibits distinct behaviour. When the hydraulic conductivity increases up to 100-fold, the concentration still gradually decreases from the hydrogel-tissue interface towards the brain parenchyma boundary, as shown in Appendix 2, Fig. 17, producing a similar response to that in Region A. However, further elevation in conductivity markedly reduces drug levels in the traumatic region while enhancing accumulation in distal tissue during oedema, thereby increasing the average concentration in Region B, as shown in Fig. 3(b). Nevertheless, the concentration also declines more rapidly due to the unsustainable supply. Likewise, the nonuniformity of drug distribution decreases with increasing capillary wall conductivity.

Figure 3(e) shows that the therapeutic drug distribution volume decreases with increasing capillary wall hydraulic conductivity until reaching a threshold of approximately 100-fold. Beyond this point, although the distribution volume initially peaks at a higher level across the brain, it subsequently declines more rapidly due to accelerated drug loss from the hydrogel.

**Fig. 4:**
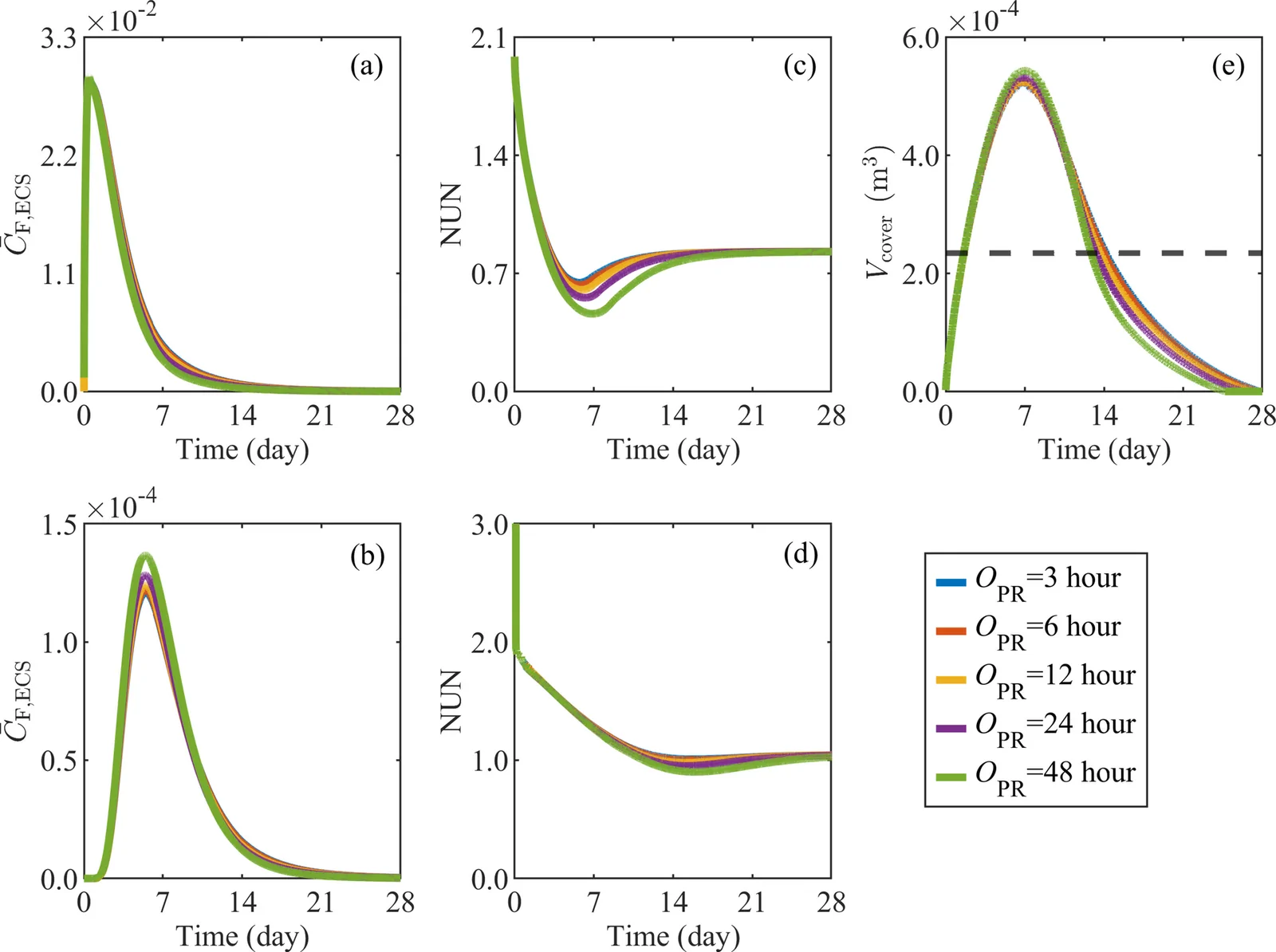
Comparison of drug delivery outcomes under oedema conditions with different peak durations. Subplots show the time course of the averaged drug concentration in Region A (**a**) and Region B (**b**); the NUN in Region A (**c**) and Region B (**d**); and the total drug-covered volume (**e**). The dark grey dashed line in subplot (e) indicates the volume of the tissue region within 2 cm of the tumour. The initial NUN in Region B can be high and fluctuating due to the extremely low concentration and heterogeneous distribution. The y-axis scale is adjusted to highlight the period when the NUN becomes relatively stable

Figure 4 compares drug delivery outcomes under oedema conditions with different peak periods. The results show that longer peak periods of oedema lead to a slightly faster reduction in drug concentration in Region A, primarily due to enhanced interstitial fluid flow. Consequently, drug concentration in the tissue Region B increases. This results in a more uniform drug distribution in both regions. Moreover, the therapeutic drug distribution volume reaches a higher peak as the peak period increases, but declines more rapidly afterwards due to greater drug loss.

**Fig. 5:**
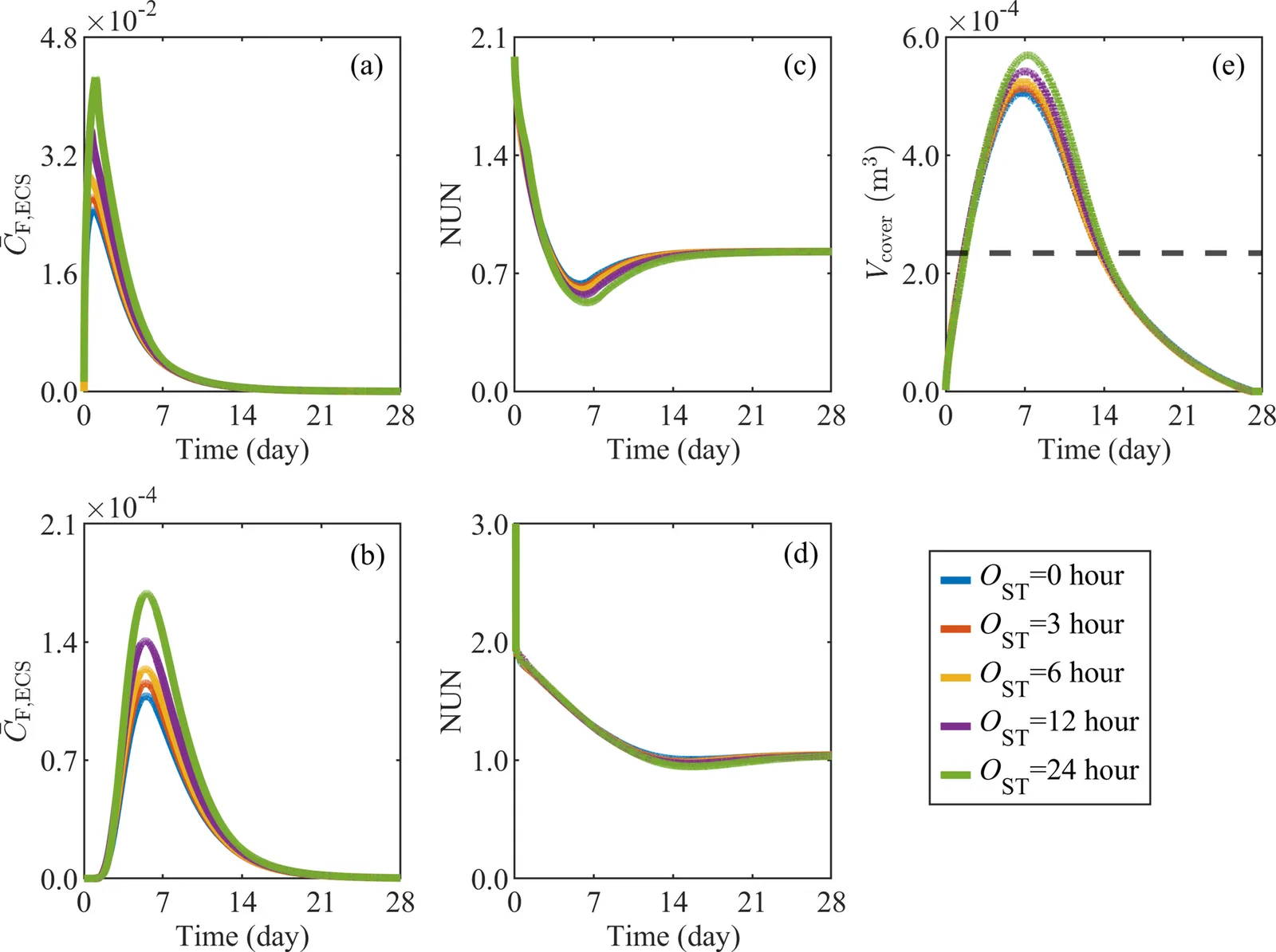
Comparison of drug delivery outcomes under oedema conditions starting at different time points after surgery. Subplots show the time course of the averaged drug concentration in Region A (**a**) and Region B (**b**); the NUN in Region A (**c**) and Region B (**d**); and the total drug-covered volume (**e**). The dark grey dashed line in subplot (e) indicates the volume of the tissue region within 2 cm of the tumour. The initial NUN in Region B can be high and fluctuating due to the extremely low concentration and heterogeneous distribution. The y-axis scale is adjusted to highlight the period when the NUN becomes relatively stable

Figure 5 presents the time courses of drug delivery outcomes under oedema conditions initiated at different time points after surgery. The modelling results indicate that delaying the onset of oedema increases the drug concentration. This effect may be attributed to the prolonged maintenance of the concentration gradient across the hydrogel-tissue interface caused by delayed oedema, which facilitates greater drug transport from the hydrogel into the brain tissue. Consequently, this leads to enhanced coverage volume and improved uniformity of drug distribution throughout the brain.

### Impact of hydrogel property

**Fig. 6:**
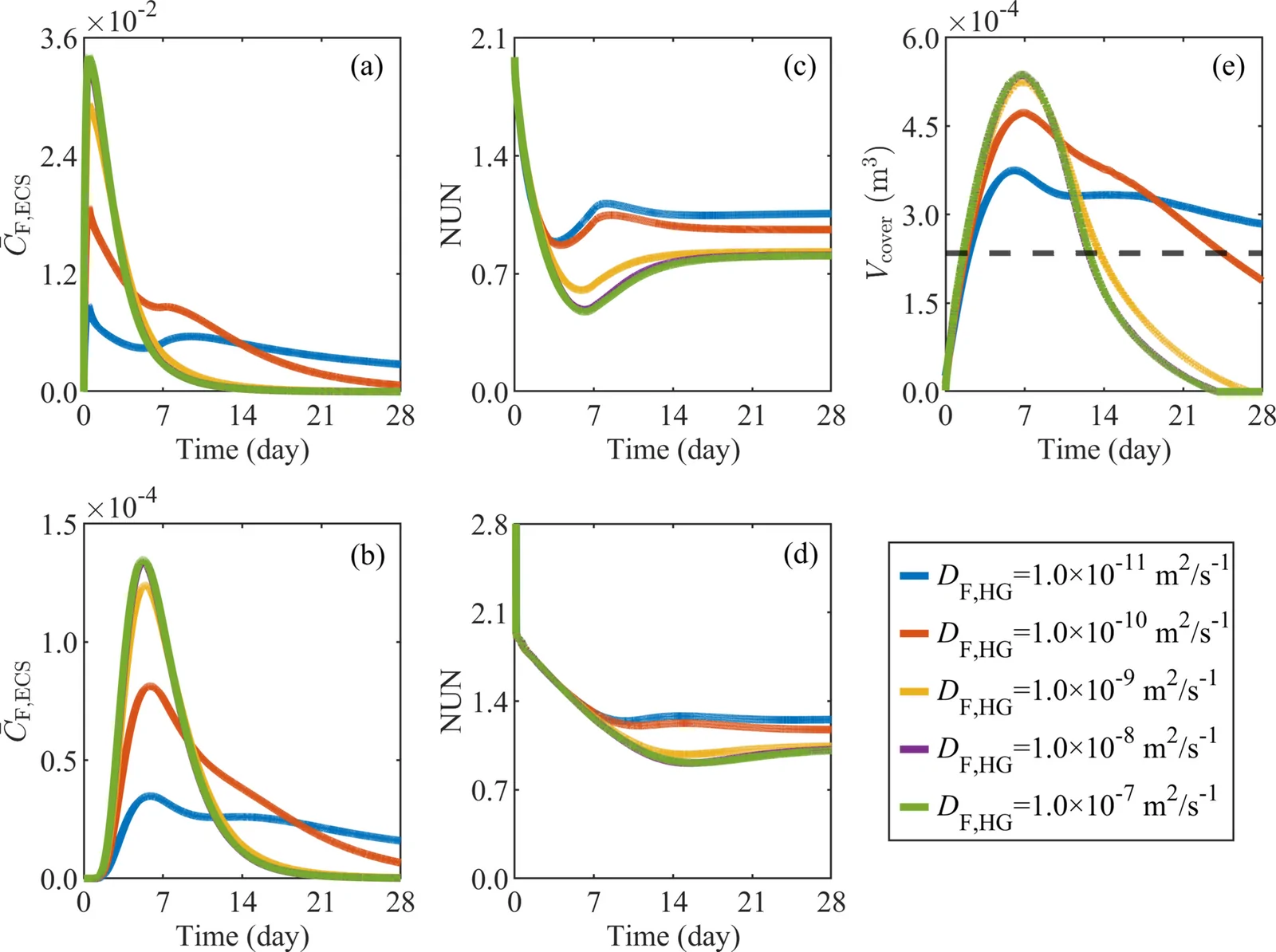
Comparison of the drug delivery profiles in hydrogels with varying drug diffusion coefficients. Subplots show the time course of the averaged drug concentration in Region A (**a**) and Region B (**b**); the NUN in Region A (**c**) and Region B (**d**); and the total drug-covered volume (**e**). The dark grey dashed line in subplot (e) indicates the volume of the tissue region within 2 cm of the tumour. The initial NUN in Region B can be high and fluctuating due to the extremely low concentration and heterogeneous distribution. The y-axis scale is adjusted to highlight the period when the NUN becomes relatively stable

Figure 6 displays the delivery outcomes obtained using hydrogels with different drug diffusion coefficients. The results indicate that increasing the diffusion coefficient accelerates drug transport from the hydrogel into the surrounding brain tissue. This leads to a higher peak concentration and a more uniform spatial distribution of the drug throughout the brain. However, the drug concentration declines more rapidly due to the limited and unsustainable release from the hydrogel. In contrast, slower drug diffusion within the hydrogel prolongs the release process, resulting in a durable, therapeutic distribution volume.

**Fig. 7:**
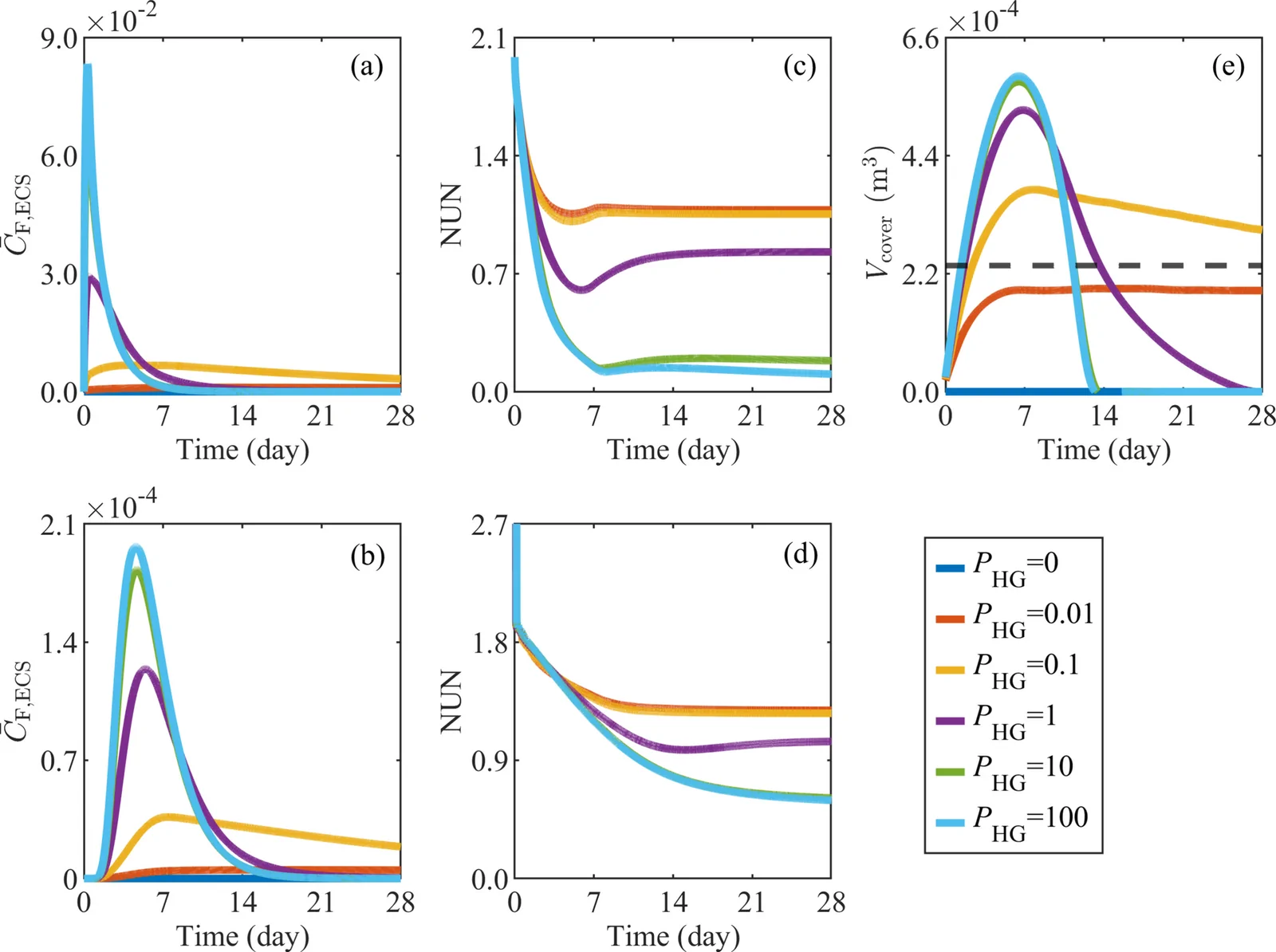
Comparison of drug delivery outcomes in hydrogels with different drug partition coefficients. Subplots show the time course of the averaged drug concentration in Region A (**a**) and Region B (**b**); the NUN in Region A (**c**) and Region B (**d**); and the total drug-covered volume (**e**). The dark grey dashed line in subplot (e) indicates the volume of the tissue region within 2 cm of the tumour. The initial NUN in Region B can be high and fluctuating due to the extremely low concentration and heterogeneous distribution. The y-axis scale is adjusted to highlight the period when the NUN becomes relatively stable

Figure 7 presents the time courses of drug delivery outcomes using hydrogels with different drug partition coefficients. The results show that increasing this partition coefficient effectively accelerates drug transfer from the hydrogel into the surrounding brain tissue, resulting in a higher peak drug concentration and a more homogeneous spatial distribution across the entire brain. Consequently, the therapeutic distribution volume also reaches a higher peak but declines to zero more rapidly due to the faster depletion of the drug source. In contrast, a lower partition coefficient enables more sustained drug release from the hydrogel. Notably, when the partition coefficient is 0.01, the therapeutic distribution volume remains confined within the tissue region located within 2 cm of the tumour margin, highlighting the potential to fine-tune this parameter to restrict drug exposure to the target region and minimise unintended effects on distant healthy brain tissue.

### Impact of drug properties

**Fig. 8:**
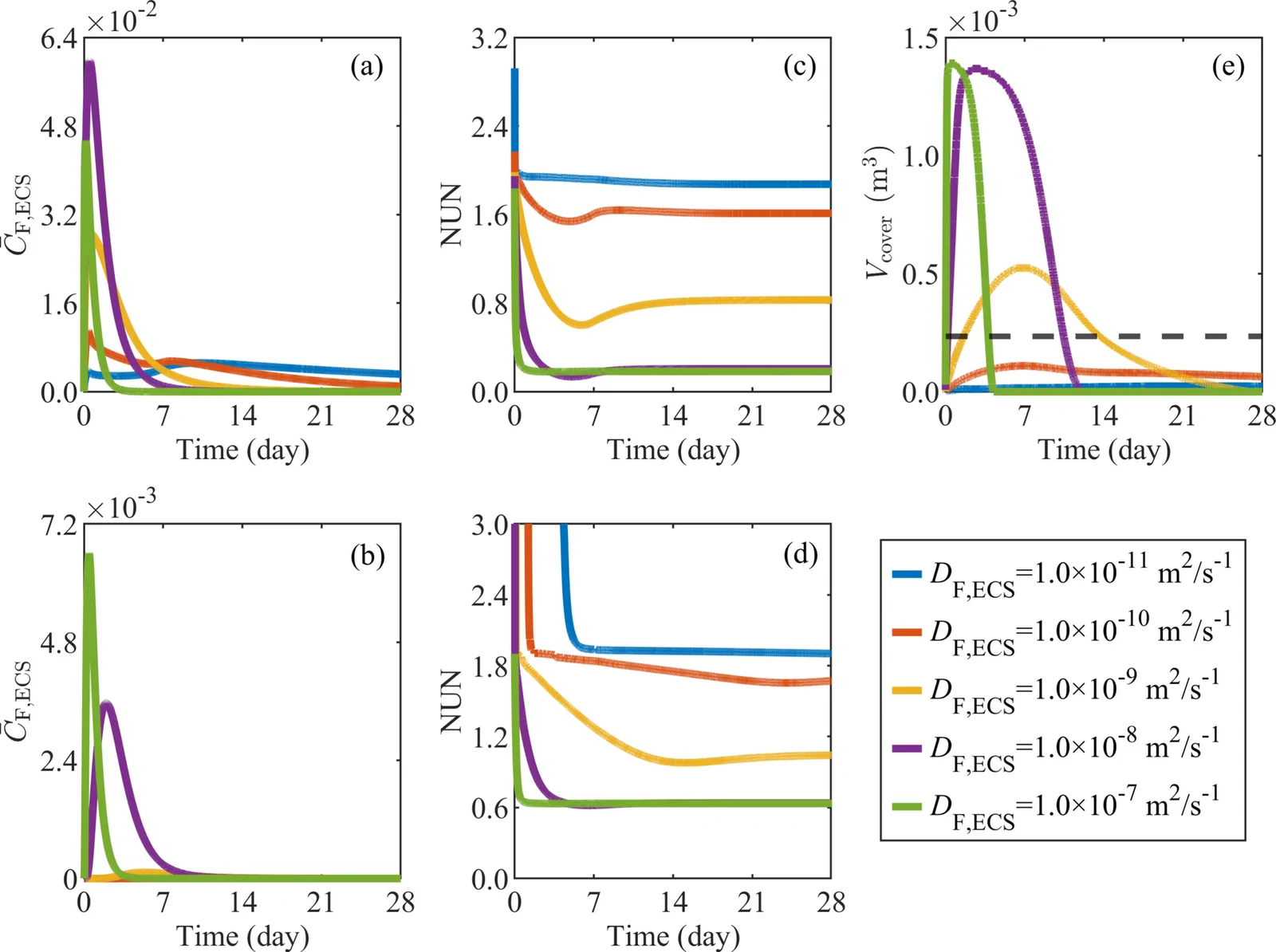
Comparison of the delivery performance of drugs with different diffusion coefficients. Subplots show the time course of the averaged drug concentration in Region A (**a**) and Region B (**b**); the NUN in Region A (**c**) and Region B (**d**); and the total drug-covered volume (**e**). The dark grey dashed line in subplot (e) indicates the volume of the tissue region within 2 cm of the tumour. The initial NUN in Region B can be high and fluctuating due to the extremely low concentration and heterogeneous distribution. The y-axis scale is adjusted to highlight the period when the NUN becomes relatively stable

Figure 8(a) shows the time course of the predicted drug concentration in Region A. The results indicate that increasing the drug diffusivity in the tissue up to a threshold of $1.0\times {10}^{-8} {\mathrm{m}}^{2}/\mathrm{s}$ effectively accelerates drug transport, resulting in a higher peak concentration. However, the concentration subsequently declines more rapidly due to the limited and unsustainable drug supply from the implanted hydrogel. When the diffusivity exceeds this threshold, the peak concentration becomes lower but still decreases rapidly in this region, as more drugs penetrate into the distal region before accumulating locally. Moreover, as shown in Fig. 8(c), increasing diffusivity results in a more uniform drug distribution, as the drug can diffuse more efficiently through the brain tissue. In contrast, the trends of drug concentration and spatial distribution exhibit a monotonic correlation with tissue diffusivity in Region B. Specifically, higher diffusivity enhances the maximum drug concentration but also accelerates its decay with time, as more drug reaches the distant tissue. Consequently, a higher diffusivity yields a more homogeneous spatial distribution, as demonstrated in Figs. 8(b) and (d).

Figure 8(e) illustrates the therapeutic drug distribution volume as a function of time. Increasing the diffusivity up to the threshold of $1.0\times {10}^{-8} {\mathrm{m}}^{2}/\mathrm{s}$ enlarges the treated tissue volume, though the coverage diminishes more quickly afterwards. Further increases beyond the threshold result in comparable peak volumes but an even earlier and faster decline, thereby limiting treatment effectiveness. Additionally, the results suggest that the drug distribution can be confined within 2 cm of the tumour margin by maintaining the diffusivity around or below $1.0\times {10}^{-10} {\mathrm{m}}^{2}/\mathrm{s}$.

**Fig. 9:**
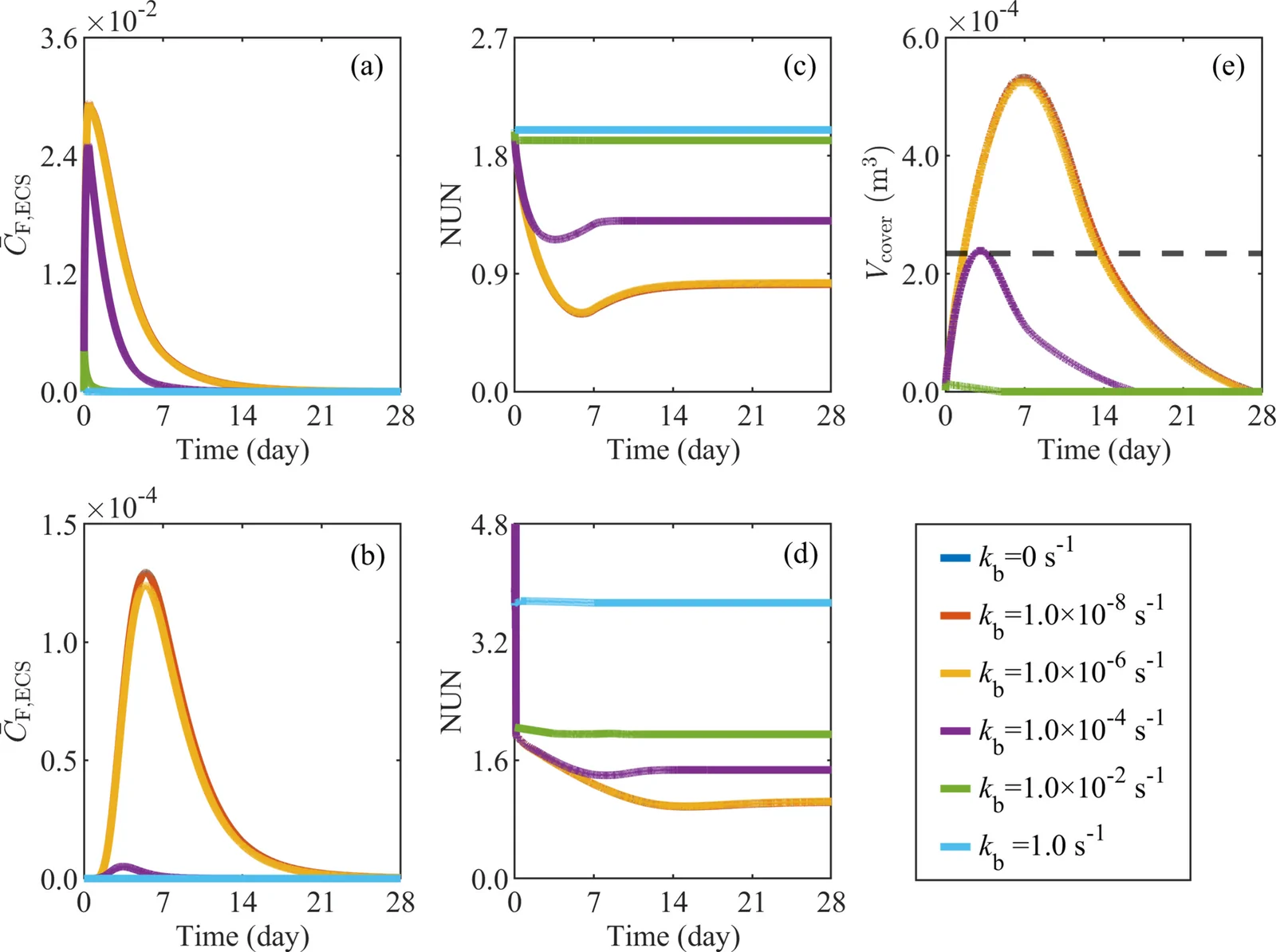
Comparison of the delivery performance of drugs under different transport rates from the tissue to the blood. Subplots show the time course of the averaged drug concentration in Region A (**a**) and Region B (**b**); the NUN in Region A (**c**) and Region B (**d**); and the total drug-covered volume (**e**). The dark grey dashed line in subplot (e) indicates the volume of the tissue region within 2 cm of the tumour. The initial NUN in Region B can be high and fluctuating due to the extremely low concentration and heterogeneous distribution. The y-axis scale is adjusted to highlight the period when the NUN becomes relatively stable

The delivery results for drugs with different transport rates from the tissue to the blood are presented in Fig. 9. As expected, reducing this rate enhances drug accumulation and distribution uniformity throughout the brain, since less drug is cleared into the bloodstream. This reduction may also lower the risk of systemic cytotoxic side effects. Moreover, slower drainage rates result in a larger therapeutic drug distribution volume. It is worth noting that further reductions have a negligible effect on the overall drug concentration and spatial distribution when the rate approaches $1.0\times {{10}^{-6} \mathrm{s}}^{-1}$.

**Fig. 10:**
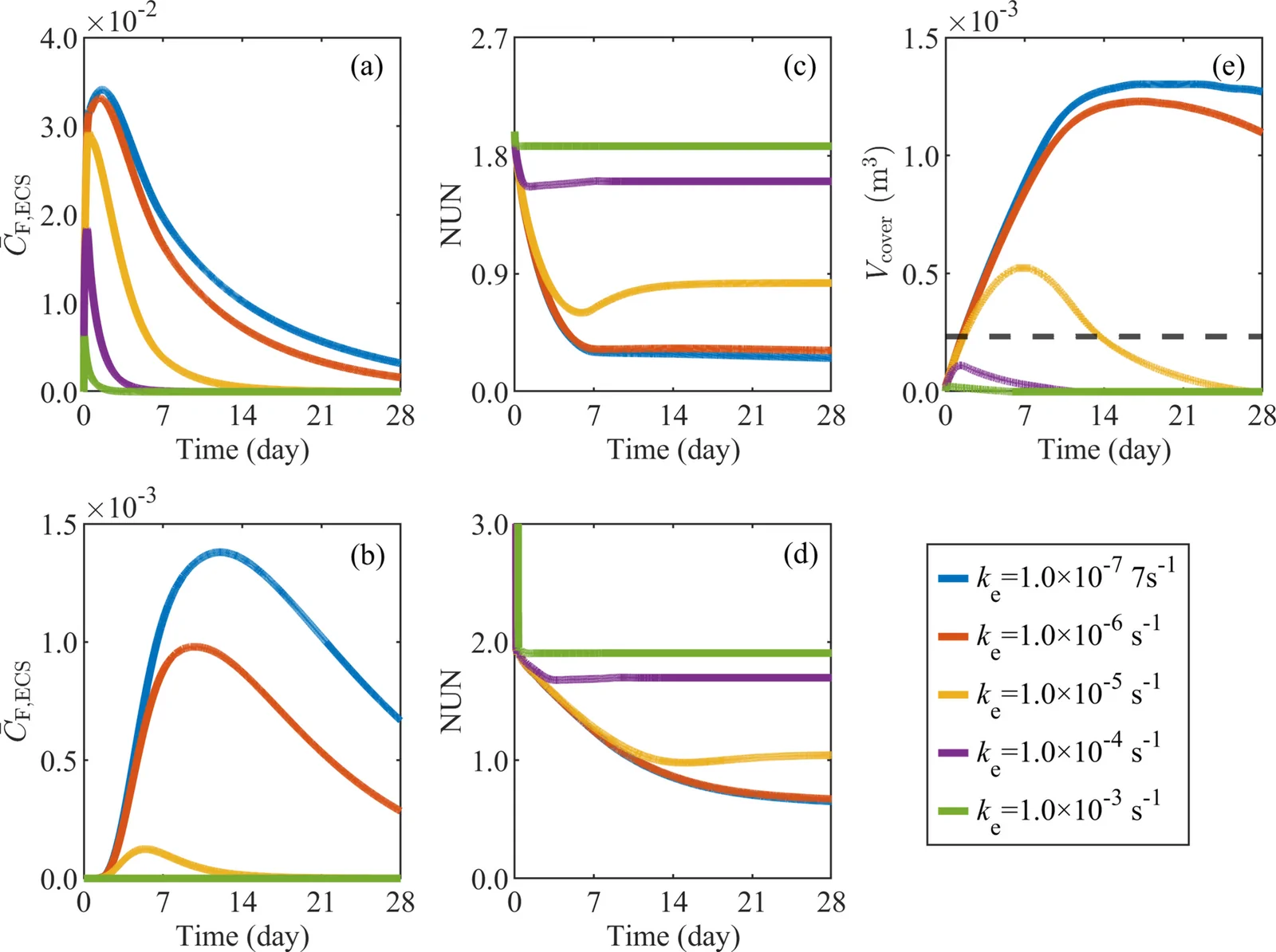
Comparison of the delivery performance of drugs under different elimination rates. Subplots show the time course of the averaged drug concentration in Region A (**a**) and Region B (**b**); the NUN in Region A (**c**) and Region B (**d**); and the total drug-covered volume (**e**). The dark grey dashed line in subplot (e) indicates the volume of the tissue region within 2 cm of the tumour. The initial NUN in Region B can be high and fluctuating due to the extremely low concentration and heterogeneous distribution. The y-axis scale is adjusted to highlight the period when the NUN becomes relatively stable

Figure 10 displays the delivery outcome using drugs with different elimination rates. Results show that using a drug with a lower elimination rate not only increases the overall drug concentration but also maintains a more sustained presence within the brain tissue. This, in turn, facilitates deeper drug penetration, leading to a more homogeneous spatial distribution and a larger distribution volume over time.

**Fig. 11:**
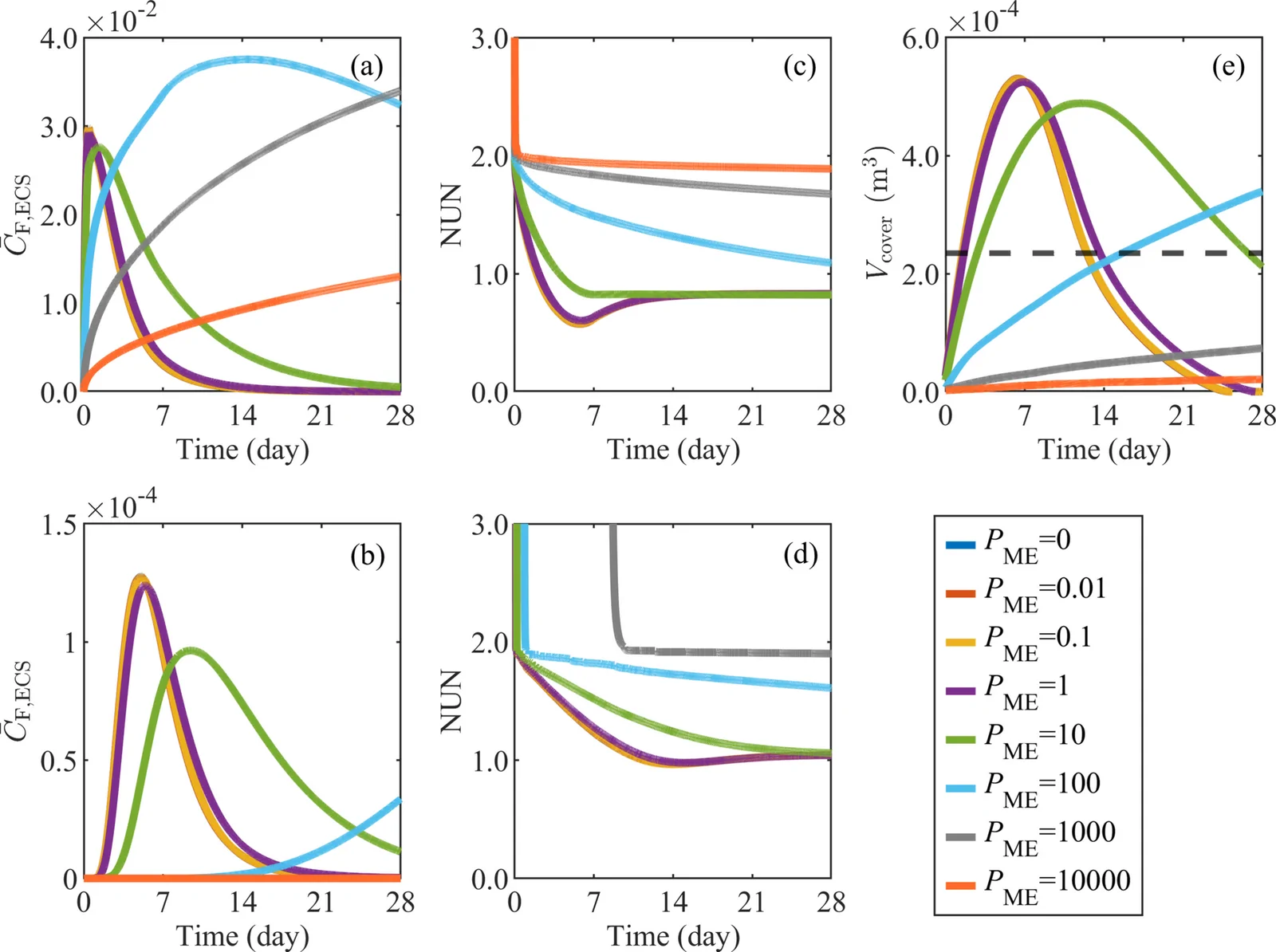
Effect of the partition coefficient between the cell membrane and tissue extracellular space on drug delivery outcomes. Subplots show the time course of the averaged drug concentration in Region A (**a**) and Region B (**b**); the NUN in Region A (**c**) and Region B (**d**); and the total drug-covered volume (**e**). The dark grey dashed line in subplot (e) indicates the volume of the tissue region within 2 cm of the tumour. The initial NUN in Region B can be high and fluctuating due to the extremely low concentration and heterogeneous distribution. The y-axis scale is adjusted to highlight the period when the NUN becomes relatively stable. Particularly, the NUN of ${P}_{\mathrm{ME}}$ in Region B remain at high levels, which is beyond the plotted range

Results summarised in Fig. 11 show that increasing the drug’s extracellular partition coefficient can greatly slow down the rate of change in drug concentration within the brain tissue, as more drugs become associated with the cell membrane. Meanwhile, the reduced amount of drug present in the extracellular space limits its loss to the bloodstream and elimination. Consequently, as shown in Fig. 11(a), within the investigated 28-day period, a higher partition coefficient results in an increased drug concentration in Region A at later times for the coefficient is 1000 or less. However, due to the delayed drug transfer to Region B, the concentration decreases within this time frame, as illustrated in Fig. 11(b). Furthermore, the stronger membrane partition slows drug penetration into the brain tissue, resulting in a more heterogeneous spatial distribution and a smaller therapeutic drug coverage volume.

**Fig. 12:**
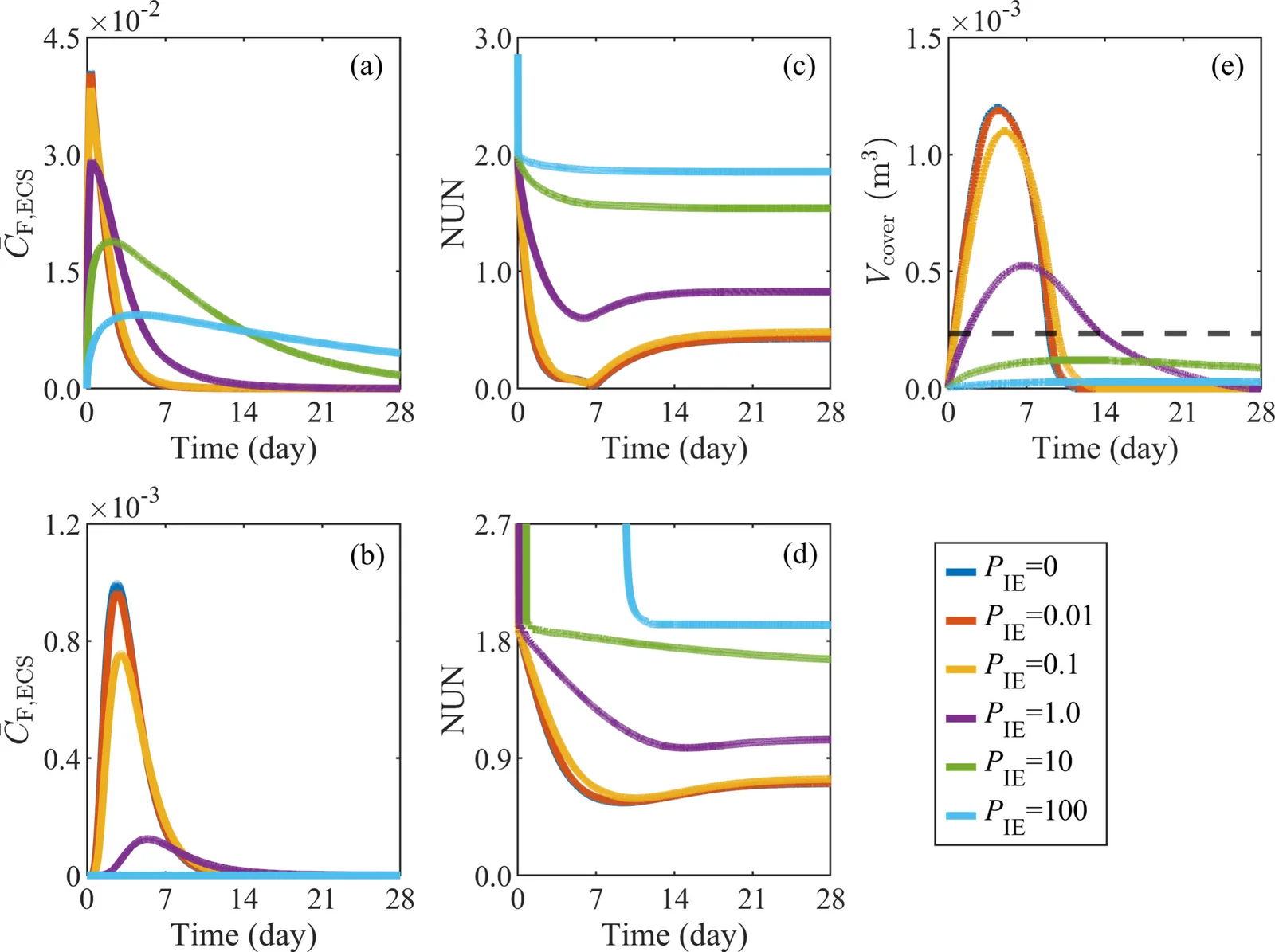
Effect of the partition coefficient between the tissue intracellular space and extracellular space on drug delivery outcomes. Subplots show the time course of the averaged drug concentration in Region A (**a**) and Region B (**b**); the NUN in Region A (**c**) and Region B (**d**); and the total drug-covered volume (**e**). The dark grey dashed line in subplot (e) indicates the volume of the tissue region within 2 cm of the tumour. The initial NUN in Region B can be high and fluctuating due to the extremely low concentration and heterogeneous distribution. The y-axis scale is adjusted to highlight the period when the NUN becomes relatively stable

Figure 12 shows the performance of drugs with different intracellular partition coefficients. The modelling predictions indicate that drugs with higher intracellular partition coefficients can substantially reduce the peak drug concentration while prolonging drug persistence within brain tissue. As a result, the spatial distribution of the drug becomes more heterogeneous. Similarly, as the partition coefficient increases, the peak value of the therapeutic drug distribution volume decreases but is maintained over a longer duration. More importantly, when this coefficient exceeds 100, the therapeutic drug distribution is largely confined to within 2 cm of the tumour margin, highlighting the potential to confine the drug distribution within Region A to reduce the risk of side effects by adjusting this parameter.

**Fig. 13:**
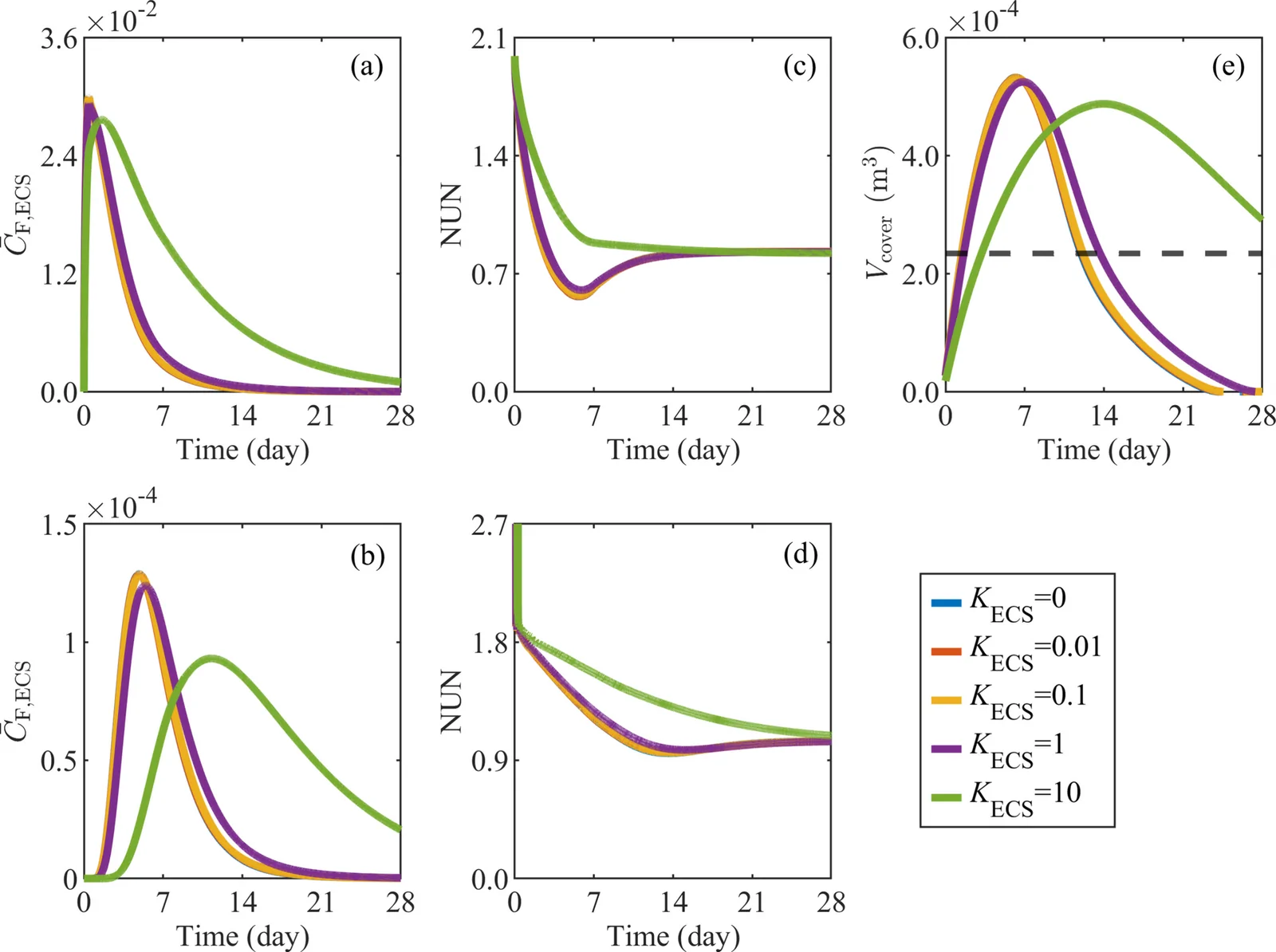
Comparison of predicted drug delivery outcomes for different extracellular binding coefficients. Subplots show the time course of the averaged drug concentration in Region A (**a**) and Region B (**b**); the NUN in Region A (**c**) and Region B (**d**); and the total drug-covered volume (**e**). The dark grey dashed line in subplot (e) indicates the volume of the tissue region within 2 cm of the tumour. The initial NUN in Region B can be high and fluctuating due to the extremely low concentration and heterogeneous distribution. The y-axis scale is adjusted to highlight the period when the NUN becomes relatively stable

Figure 13 presents the predicted delivery outcomes of drugs with different binding coefficients in the tissue extracellular space. The results demonstrate that increasing this parameter slightly reduces the peak drug concentration in Region A, yet allows the concentration to be sustained at a higher level at later times. This concentration profile results in a more heterogeneous drug distribution during the first two weeks after surgery; however, the degree of non-uniformity tends to converge for drugs with different extracellular binding coefficients. Similar trends are observed in Region B. Consequently, drugs with higher extracellular binding coefficients provide a more durable coverage, as illustrated in Fig. 13(e).

**Fig. 14:**
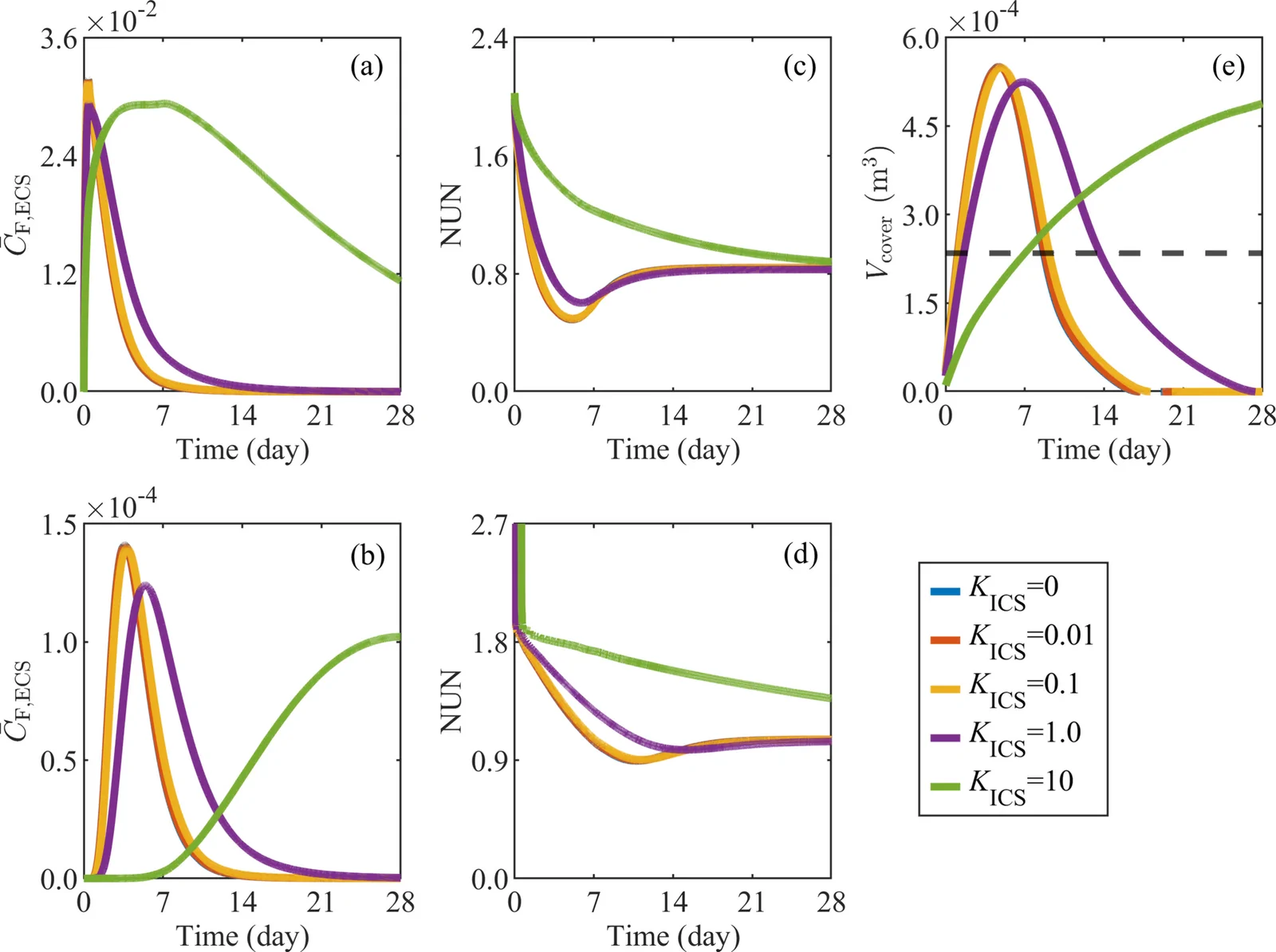
Comparison of predicted drug delivery outcomes for different intracellular binding coefficients. Subplots show the time course of the averaged drug concentration in Region A (**a**) and Region B (**b**); the NUN in Region A (**c**) and Region B (**d**); and the total drug-covered volume (**e**). The dark grey dashed line in subplot (e) indicates the volume of the tissue region within 2 cm of the tumour. The initial NUN in Region B can be high and fluctuating due to the extremely low concentration and heterogeneous distribution. The y-axis scale is adjusted to highlight the period when the NUN becomes relatively stable

Figure 14 shows the predicted delivery outcomes of drugs with different intracellular binding coefficients. The results indicate that increasing the binding coefficient markedly prolongs drug persistence in Region A, especially when the parameter exceeds 1.0. Although this leads to a less uniform drug distribution in this region, the drug is able to sustain therapeutic coverage more effectively. In contrast, drugs with higher intracellular binding coefficients delay accumulation in Region B and produce a more heterogeneous distribution in this area, as illustrated in Fig. 14(b) and (d).

### Sensitivity analysis

**Fig. 15:**
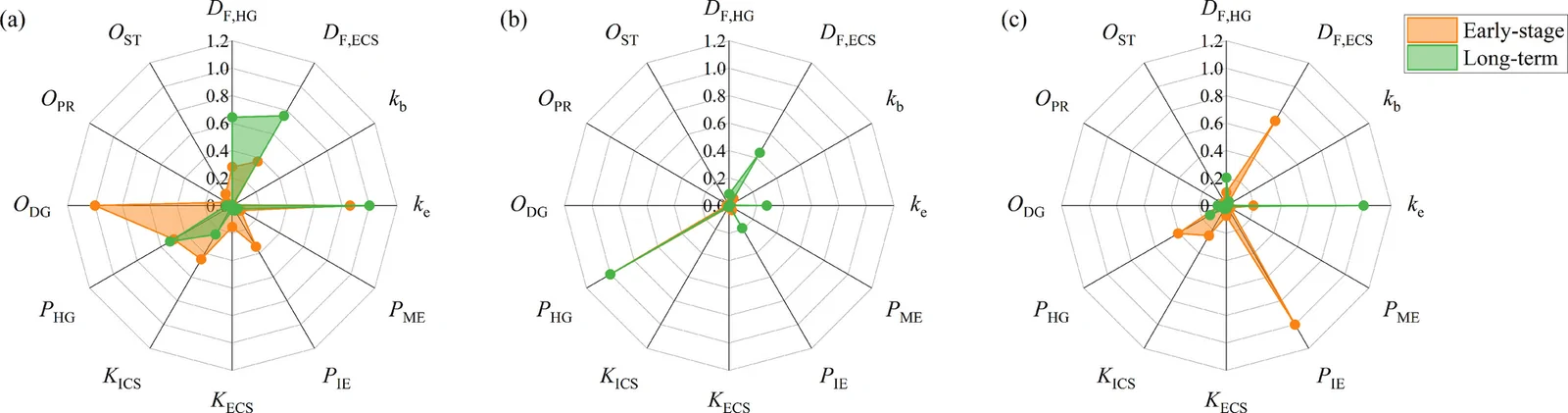
Parameter sensitivity analysis of delivery outcomes at the early stage (first 7 days) and in the long term (last 7 days). The impact is evaluated by the normalised sensitivity ${\overline{S} }_{\mathrm{overall},\mathrm{i}}$, defined in Eq. (8). (**a**) drug concentration and (**b**) distribution non-uniformity in Region A; (**c**) therapeutic distribution volume

**Fig. 16:**
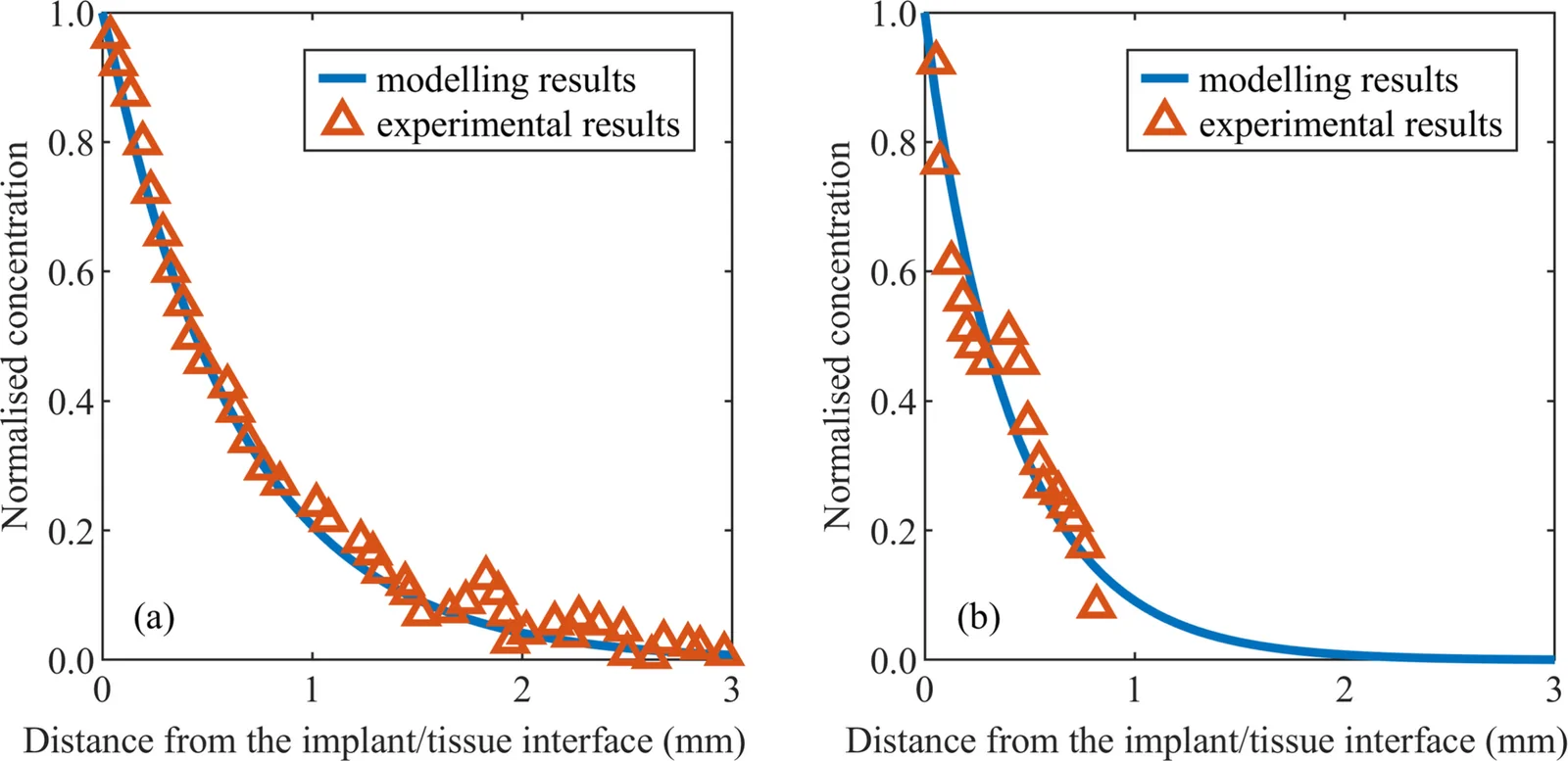
Model validation. Comparison between model-predicted and experimentally measured carmustine concentrations as a function of penetration depth in brain tissue on Day 3 (**a**) and Day 14 (**b**). The experimental data and modelling parameters are obtained from Ref. [27]

**Fig. 17:**
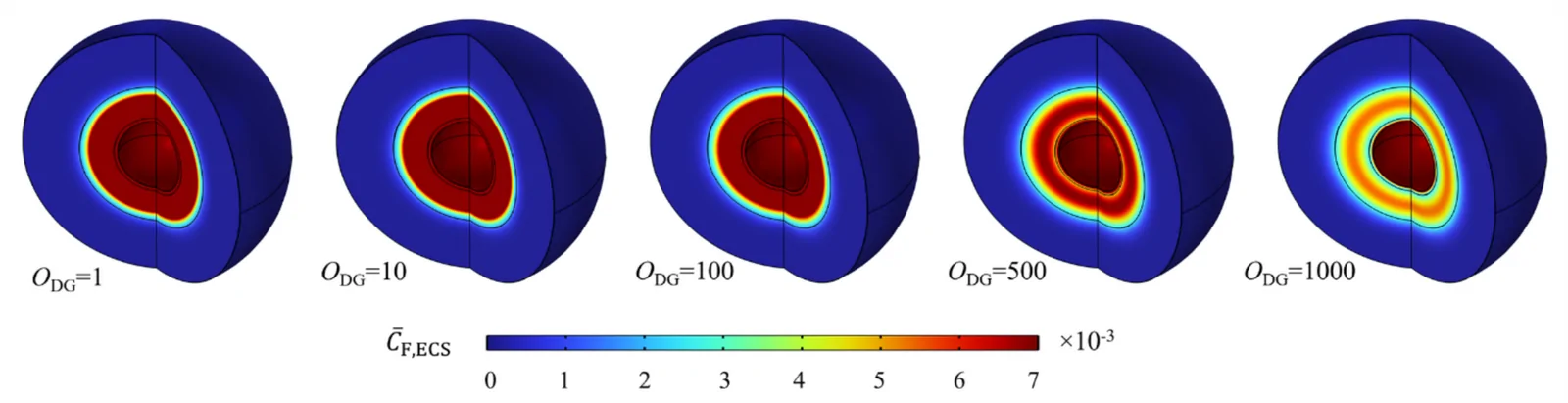
Spatial distribution of free drugs in the brain tissues under different hydraulic conductivities of traumatic blood capillaries at 4 h, during the oedema phase

A sensitivity analysis is performed to compare the impact of the 12 examined tissue and drug properties on drug concentration and distribution heterogeneity in Region A, where over 90% of tumour recurrence occurs, as well as on the therapeutic distribution volume during the first 7 days (early stage) and the last 7 days (long term). As illustrated in Fig. 15, drug concentration at the early stage is predominantly governed by the degree of oedema, followed by the drug elimination rate in the tissue. In contrast, drug accumulation in the long term is primarily determined by the elimination rate, with diffusivity in the brain tissue and the hydrogel ranking second and third, respectively. Moreover, distribution heterogeneity at both the early stage and in the long term is mainly controlled by the partition coefficient between the hydrogel and the tissue. Unlike the early-stage behaviour, diffusivity in the tissue, the elimination rate, and the partition coefficient between the intracellular and extracellular spaces also contribute to distribution heterogeneity in the long term. With respect to the therapeutic distribution volume, the partition coefficient between the intracellular and extracellular spaces plays the dominant role at the early stage, followed by diffusivity in the brain tissue and partition coefficient between the hydrogel and the tissue. In contrast, the therapeutic distribution volume in the long term is more strongly influenced by the drug elimination rate.

## Discussion

The hydrogel implemented in the surgery left cavity can directly deliver the loaded drug to its surrounding brain tissues and result in a high enough concentration for effectively killing the residual cells. The drug accumulation and distribution are strongly influenced by the oedema, which can accelerate the interstitial fluid flow to promote the drug’s convective transport for a more homogeneous distribution.

The responses of drug delivery outcomes to variations in hydrogel properties, drug properties, and physiological processes exhibit substantial differences. These variations highlight the potential for enhancing therapeutic efficacy through systematic optimisation of the delivery system design. For example, employing hydrogels with higher permeability or a lower partition coefficient of the encapsulated drug towards the hydrogel phase can enhance therapeutic efficacy over a larger tissue region within a relatively short period following administration; however, the therapeutic effect tends to decline more rapidly at later times. The selection of the loaded drug also plays a crucial role. Drugs with low permeability across capillary walls and limited reactivity within the tissue microenvironment can substantially improve drug accumulation and spatial distribution, thereby promoting better therapeutic outcomes. Moreover, hydrophobic drugs or those with strong binding affinities to extracellular or intracellular components can achieve more sustained therapeutic effects across broader tissue regions, as these components, including cell membranes, may act as local reservoirs that retard drug clearance, albeit at the expense of spatial uniformity. In addition, drugs with low intracellular partition coefficients tend to achieve higher concentrations, more homogeneous distributions, and larger therapeutic diffusion volumes. Nevertheless, an optimal range of extracellular diffusivity likely exists to maximise drug accumulation and dispersion, indicating that the molecular weight and surface charge of the drug should be carefully tailored to achieve effective delivery performance. The sensitivity analysis demonstrates the key role of the drug elimination rate and diffusivity in determining drug concentration and therapeutic distribution volume. As an intrinsic drug property, this finding highlights the importance of selecting an appropriate drug for hydrogel loading. Similar observations are found for the hydrogel-tissue partition coefficient, further emphasising the role of drug-material interactions in governing delivery outcomes.

Translation of these findings into practical hydrogel design can be achieved by focusing on a small number of experimentally controllable parameters with strong influence on delivery. In particular, drug diffusivity in hydrogel and hydrogel-tissue partition coefficient are highly amenable to experimental tuning through polymer concentration, crosslinking density, and chemical functionalisation. Increasing drug diffusivity in hydrogel or reducing affinity enhances early drug release and spatial coverage, whereas stronger interactions prolong retention and sustain exposure. Drug-related properties such as intracellular partitioning and binding are intrinsic, but they can inform drug or carrier selection. Although post-operative oedema itself cannot be controlled by the hydrogel, its strong influence on delivery suggests that release kinetics should be designed to remain effective across variable oedema onset and duration.

In post-surgical glioblastoma patients, the onset and duration of vasogenic oedema are highly variable, influenced by surgical trauma, tumour location, and patient-specific factors. Clinically, corticosteroids such as dexamethasone are administered to stabilise the blood–brain barrier and reduce oedema, yet the timing and dose can differ between patients, resulting in dynamic interstitial fluid flows that affect local drug transport. Our modelling results indicate that both the onset and persistence of oedema can substantially alter the spatial distribution and therapeutic volume of hydrogel-released drugs. Early or prolonged oedema can enhance initial drug penetration and homogenise distribution, whereas delayed oedema may sustain drug exposure over a larger tissue volume. These insights highlight the need to consider patient-specific oedema dynamics and steroid management when designing hydrogel formulations and dosing schedules, thereby improving the translational applicability of local post-operative drug delivery strategies.

The model employed in this study was developed to capture the key processes involved in hydrogel-mediated drug delivery to brain tissue. It has been previously validated against reported experimental data [31]. However, it is important to note that such modelling primarily provides qualitative predictions, which are more suitable for cross-comparative analyses to identify the influence of specific factors rather than for direct quantitative forecasting. At the same time, some assumptions and limitations should be acknowledged. First, a 2-D axisymmetric configuration is adopted in this study instead of a fully 3-D brain geometry, while maintaining physiologically realistic model dimensions. This simplification is necessary because simulations based on a 3-D geometry require substantial computational resources, making large-scale sensitivity analyses across multiple parameters computationally prohibitive. Additional simulations demonstrate that predictions obtained using the 2-D axisymmetric geometry qualitatively agree with those generated using a 3-D realistic brain geometry reconstructed from patient medical images. As the primary objective of this study is to investigate the influence of individual tissue and drug properties based on qualitative comparisons of trends across simulations, the 2-D axisymmetric geometry is sufficient for the purposes of the present work. In future studies, realistic brain geometries and tissue properties reconstructed from medical imaging data may be employed to enable patient-specific simulations requiring quantitative predictions [49]. Second, the processes of drug binding and cellular uptake were simplified by assuming instantaneous dynamic equilibrium, as the time scales of these processes are much shorter than the overall time frame of drug delivery. More detailed models could be incorporated to capture their transient behaviours where necessary [50–52]. Third, a uniform threshold is adopted in this study to evaluate drug coverage, enabling direct comparison of delivery outcomes across different delivery scenarios on a consistent basis. However, it should be noted that the choice of threshold may vary, particularly in drug-specific studies, where the coverage volume may alternatively be defined using pharmacologically relevant metrics, such as the LD₉₀ of the studied drug under consideration. Fourth, the present model focuses exclusively on drug transport, accumulation, and distribution, without explicitly incorporating cell-killing dynamics. This choice is motivated by the fact that the drug properties explored in the parametric study correspond to different classes of drugs, whereas pharmacodynamic models, including their governing formulations and parameter values, vary considerably among drug types. As a result, adopting a single, unified pharmacodynamic model would be inappropriate for the scope of the present work. In future studies, pharmacodynamic model [53–57] and tumour growth model can be integrated for drug-specific investigations, in which model parameters should be experimentally determined to ensure accurate predictions of tumour cell survival or recurrence rates.

## Conclusions

A comprehensive parametric study is conducted to investigate the effects of various hydrogel, drug, and tissue properties on hydrogel-mediated drug delivery to the brain following tumour resection, using mathematical modelling. The results demonstrate that hydrogels can effectively administer the encapsulated drugs to surrounding tissues; however, the delivery outcomes are strongly influenced by the post-surgical oedema. Specifically, although a longer duration and delayed onset of oedema both contribute to a more homogeneous drug distribution, the former produces a slightly larger but faster-declining therapeutic drug volume, whereas the latter yields a larger and more sustained therapeutic drug volume. Hydrogels that are more permeable or have lower affinity for the encapsulated drugs not only promote a more homogeneous distribution over time but also enhance the drug concentration and therapeutic distribution volume at early stages following implantation; nevertheless, these effects diminish more rapidly with time. Drugs with lower intracellular partitioning can lead to higher concentrations, more homogeneous distributions, and larger therapeutic distribution volumes during the early delivery phase. In contrast, drugs with stronger binding affinities to extracellular or intracellular components, or a higher tendency to associate with cell membranes, can sustain therapeutic concentrations for longer durations. Furthermore, drugs with lower transvascular permeability or slower elimination rates contribute to improved delivery outcomes, while the drug diffusivity in the extracellular space should be optimised to achieve the maximum drug concentration and therapeutic distribution volume. The findings from this study provide valuable insights for optimising the design of hydrogel-based drug delivery systems to prevent glioblastoma recurrence in the brain.

## Data Availability

The datasets generated during and/or analysed during the current study are available from the corresponding author on reasonable request.

## Appendix 1

**Table 2:** Governing equations of the mathematical model [31]

1	Fluid transport model	
	$$\left\{\begin{array}{c}\nabla \bullet u={F}_{\mathrm{b}}=L\frac{S}{V}\left({p}_{\mathrm{b}}-{p}_{\mathrm{i}}-\sigma \Delta \pi \right)\\ \rho \left(\frac{\partial \mathbf{u}}{\partial t}+\mathbf{u}\bullet \nabla \mathbf{u}\right)=-\nabla {p}_{\mathrm{i}}+\mu {\nabla }^{2}u-\frac{\mu }{\kappa }u\end{array}\right.$$	(A1)
2	Drug transport model in the hydrogel	
	$$\frac{\partial {C}_{\mathrm{F},\mathrm{HG}}}{\partial t}=\nabla \bullet \left({D}_{\mathrm{F},\mathrm{HG}}\nabla {C}_{\mathrm{F},\mathrm{HG}}\right)$$	(A2)
3	Drug transport model in the brain tissue	
	$$\frac{\partial {C}_{\mathrm{F},\mathrm{ECS}}}{\partial t}=\nabla \bullet \left({D}_{\mathrm{F},\mathrm{ECS}}^{*}\nabla {C}_{\mathrm{F},\mathrm{ECS}}\right)-{\mathbf{u}}^{\boldsymbol{*}}\bullet \nabla {C}_{\mathrm{F},\mathrm{ECS}}-{k}_{\mathrm{e}}^{*}{C}_{\mathrm{F},\mathrm{ECS}}$$	(A3)
where$\mathbf{u}$is the interstitial fluid flow velocity, and${p}_{\mathrm{i}}$is the pressure.${C}_{\mathrm{F},\mathrm{HG}}$and${C}_{\mathrm{F},\mathrm{ECS}}$are the concentration of free drugs in the tissue extracellular space and intracellular space, respectively. The drug’s apparent diffusivity is${D}_{\mathrm{F},\mathrm{ECS}}^{*}=\left(\alpha /m\right){D}_{\mathrm{F},\mathrm{ECS}}$; the apparent interstitial fluid flow velocity${\mathbf{u}}^{\boldsymbol{*}}=\left(\alpha /m\right)\mathbf{u}$; the drug’s apparent removal rate${k}_{\mathrm{e}}^{*}=\left[\alpha {k}_{\mathrm{b}}+\left(\alpha +{P}_{\mathrm{IE}}\gamma \right){k}_{\mathrm{e}}+{F}_{\mathrm{b}}\right]/m$;$m=\alpha \left(1+{K}_{\mathrm{ECS}}\right)+\gamma {P}_{\mathrm{IE}}\left(1+{K}_{\mathrm{ICS}}\right)+\beta {P}_{\mathrm{ME}}$. The rest model parameters and their values are defined in Section "Model parameters"		

The model-predicted carmustine concentration profiles from the gel-tissue interface into the brain parenchyma are compared with the experimental data in Fig. 16. The good agreement between the predictions and measurements supports the validity of the model.

## Appendix 2

Figure 17 illustrates the spatial distribution of the drug under varying hydraulic conductivities of traumatic blood capillaries at 4 h. Although the drug concentration in Region A decreases with increasing hydraulic conductivity of the blood vessels, the overall distribution pattern transitions from a continuous decline from the hydrogel towards the brain parenchyma surface to a distinct accumulation within Region A, thereby resulting in an elevated concentration in Region B.
